# Diagnostic Accuracy of a Plasma Phosphorylated Tau 217 Immunoassay for Alzheimer Disease Pathology

**DOI:** 10.1001/jamaneurol.2023.5319

**Published:** 2024-01-22

**Authors:** Nicholas J. Ashton, Wagner S. Brum, Guglielmo Di Molfetta, Andrea L. Benedet, Burak Arslan, Erin Jonaitis, Rebecca E. Langhough, Karly Cody, Rachael Wilson, Cynthia M. Carlsson, Eugeen Vanmechelen, Laia Montoliu-Gaya, Juan Lantero-Rodriguez, Nesrine Rahmouni, Cecile Tissot, Jenna Stevenson, Stijn Servaes, Joseph Therriault, Tharick Pascoal, Alberto Lleó, Daniel Alcolea, Juan Fortea, Pedro Rosa-Neto, Sterling Johnson, Andreas Jeromin, Kaj Blennow, Henrik Zetterberg

**Affiliations:** 1 Department of Psychiatry and Neurochemistry, Institute of Neuroscience & Physiology, Sahlgrenska Academy at the University of Gothenburg, Mölndal, Sweden; 2 King’s College London, Institute of Psychiatry, Psychology and Neuroscience Maurice Wohl Institute Clinical Neuroscience Institute, London, United Kingdom; 3 NIHR Biomedical Research Centre for Mental Health and Biomedical Research Unit for Dementia at South London and Maudsley NHS Foundation, London, United Kingdom; 4 Centre for Age-Related Medicine, Stavanger University Hospital, Stavanger, Norway; 5 Graduate Program in Biological Sciences: Biochemistry, Universidade Federal do Rio Grande do Sul (UFRGS), Porto Alegre, Brazil; 6 Wisconsin Alzheimer’s Institute, School of Medicine and Public Health, University of Wisconsin–Madison, Madison; 7 Wisconsin Alzheimer’s Disease Research Center, School of Medicine and Public Health, University of Wisconsin–Madison, Madison; 8 Department of Medicine, Division of Geriatrics and Gerontology, School of Medicine and Public Health, University of Wisconsin–Madison, Madison; 9 Geriatric Research Education and Clinical Center of the Wm. S. Middleton Memorial Veterans Hospital, Madison, Wisconsin; 10 ADx NeuroSciences, Ghent, Belgium; 11 Translational Neuroimaging Laboratory, McGill University Research Centre for Studies in Aging, Alzheimer’s Disease Research Unit, Douglas Research Institute, Le Centre Intégré Universitaire de Santé et de Services Sociaux (CIUSSS) de l’Ouest-de-l’Île-de-Montréal; 12 Department of Neurology and Neurosurgery, Psychiatry and Pharmacology and Therapeutics, McGill University, Montreal, Quebec, Canada; 13 Department of Psychiatry, University of Pittsburgh, Pittsburgh, Pennsylvania; 14 Department of Neurology, University of Pittsburgh, Pittsburgh, Pennsylvania; 15 Department of Neurology, Institut d’Investigacions Biomèdiques Sant Pau, Hospital de Sant Pau, Universitat Autònoma de Barcelona, Hospital de la Santa Creu i Sant Pau, Barcelona, Catalunya; 16 Centro de Investigación Biomédica en Red en Enfermedades Neurodegenerativas, CIBERNED, Madrid, Spain; 17 ALZpath, Carlsbad, California; 18 Clinical Neurochemistry Laboratory, Sahlgrenska University Hospital, Mölndal, Sweden; 19 Department of Neurodegenerative Disease, UCL Institute of Neurology, London, United Kingdom; 20 UK Dementia Research Institute at UCL, London, United Kingdom; 21 Hong Kong Center for Neurodegenerative Diseases, Clear Water Bay, Hong Kong, China

## Abstract

**Importance:**

Phosphorylated tau (p-tau) is a specific blood biomarker for Alzheimer disease (AD) pathology, with p-tau217 considered to have the most utility. However, availability of p-tau217 tests for research and clinical use has been limited. Expanding access to this highly accurate AD biomarker is crucial for wider evaluation and implementation of AD blood tests.

**Objective:**

To determine the utility of a novel and commercially available immunoassay for plasma p-tau217 to detect AD pathology and evaluate reference ranges for abnormal amyloid β (Aβ) and longitudinal change across 3 selected cohorts.

**Design, Setting, and Participants:**

This cohort study examined data from 3 single-center observational cohorts: cross-sectional and longitudinal data from the Translational Biomarkers in Aging and Dementia (TRIAD) cohort (visits October 2017–August 2021) and Wisconsin Registry for Alzheimer’s Prevention (WRAP) cohort (visits February 2007–November 2020) and cross-sectional data from the Sant Pau Initiative on Neurodegeneration (SPIN) cohort (baseline visits March 2009–November 2021). Participants included individuals with and without cognitive impairment grouped by amyloid and tau (AT) status using PET or CSF biomarkers. Data were analyzed from February to June 2023.

**Exposures:**

Magnetic resonance imaging, Aβ positron emission tomography (PET), tau PET, cerebrospinal fluid (CSF) biomarkers (Aβ42/40 and p-tau immunoassays), and plasma p-tau217 (ALZpath pTau217 assay).

**Main Outcomes and Measures:**

Accuracy of plasma p-tau217 in detecting abnormal amyloid and tau pathology, longitudinal p-tau217 change according to baseline pathology status.

**Results:**

The study included 786 participants (mean [SD] age, 66.3 [9.7] years; 504 females [64.1%] and 282 males [35.9%]). High accuracy was observed in identifying elevated Aβ (area under the curve [AUC], 0.92-0.96; 95% CI, 0.89-0.99) and tau pathology (AUC, 0.93-0.97; 95% CI, 0.84-0.99) across all cohorts. These accuracies were comparable with CSF biomarkers in determining abnormal PET signal. The detection of abnormal Aβ pathology using a 3-range reference yielded reproducible results and reduced confirmatory testing by approximately 80%. Longitudinally, plasma p-tau217 values showed an annual increase only in Aβ-positive individuals, with the highest increase observed in those with tau positivity.

**Conclusions and Relevance:**

This study found that a commercially available plasma p-tau217 immunoassay accurately identified biological AD, comparable with results using CSF biomarkers, with reproducible cut-offs across cohorts. It detected longitudinal changes, including at the preclinical stage.

## Key Points

**Question:**

What are the capabilities of a commercially available plasma phosphorylated tau 217 (p-tau217) immunoassay to identify Alzheimer disease pathophysiology?

**Findings:**

This cohort study found that the p-tau217 immunoassay showed similar accuracies to cerebrospinal fluid biomarkers in identifying abnormal amyloid β (Aβ) and tau pathologies. A 3-range reference for detecting abnormal Aβ pathology was consistent across 3 cohorts; over 8 years, the largest change of p-tau217 was in individuals positive for both Aβ and tau.

**Meaning:**

The wider availability of high-performing assays may expedite the use of blood biomarkers in clinical settings and benefit the research community.

## Teaser

This cohort study examines data from 3 single-center observational cohorts to test the capabilities of a commercially available plasma phosphorylated tau 217 immunoassay to identify Alzheimer disease pathophysiology.

## Introduction

In Alzheimer disease (AD), blood biomarkers have emerged as scalable tools for clinical evaluation, trial recruitment, and disease monitoring.^1^ Their anticipated implementation aims to substantially reduce the reliance on cerebrospinal fluid (CSF) or positron emission tomography (PET) scans in specialized centers.^2^ Moreover, a robust and accurate blood-based biomarker would enable a more comprehensive assessment of cognitive impairment in settings where advanced testing is limited. Therefore, use of a blood biomarker is intended to enhance an early and precise AD diagnosis, leading to improved patient management and, ultimately, timely access to disease-modifying therapies.

Phosphorylated tau (p-tau) is the leading blood biomarker candidate, demonstrating superior diagnostic accuracy and disease specificity compared with other candidates.^3,4^ The amyloid β 42/40 (Aβ42/40) ratio, a validated CSF biomarker,^5^ has limitations in blood^6,7^ and lacks the robustness required for routine clinical testing.^8,9^ In contrast, high-performing p-tau blood test results exhibit a substantial increase in patients with AD,^10^ occurring concurrently with extracellular Aβ plaque deposition, an AD hallmark feature. This relationship is observed across the AD continuum, including the asymptomatic phase in sporadic and genetic forms of AD.^11,12,13,14^ Yet certain p-tau species, but not all, are also associated with neurofibrillary tangle pathology, the secondary AD pathological hallmark.^15,16,17^ Thus, p-tau is regarded as the primary blood biomarker for AD pathology throughout all stages of the disease.

Among proposed blood tau biomarkers,^18,19,20,21^ phosphorylated tau at threonine 217 (p-tau217) has consistently shown high performance in differentiating AD from other neurodegenerative disorders^10,22^ and in detecting AD pathology in patients with mild cognitive impairment (MCI).^22^ Notably, p-tau217 exhibits larger-fold changes compared with p-tau181 and p-tau231,^10^ often achieving high discrimination, with areas under the curve (AUC) exceeding 90%.^19,23^ Additionally, p-tau217 demonstrates a unique longitudinal trajectory, showing increases associated with worsening brain atrophy and declining cognitive performance in individuals with elevated Aβ pathology.^24,25^

With the imminent implementation of anti-Aβ therapies in dementia management, validated blood biomarkers are urgently needed to guide timely treatment decisions. While plasma p-tau217 has shown promise as a diagnostic tool for AD, its widespread evaluation has been hindered by limited availability of commercial assays. This study aims to address this gap by assessing the utility of ALZpath pTau217, a commercially available immunoassay, to highlight the presence of AD pathology. In addition, we aim to report reference ranges of the plasma p-tau217 that correspond to abnormal amyloid PET and CSF measures.

## Methods

This study included participants from 3 observational cohorts: the Translational Biomarkers in Aging and Dementia (TRIAD), Wisconsin Registry for Alzheimer’s Prevention (WRAP), and Sant Pau Initiative on Neurodegeneration (SPIN). Participants gave written or verbal informed consent, and the studies were approved by the relevant ethics boards (eMethods in Supplement 1). The present study followed the Strengthening the Reporting of Observational Studies in Epidemiology (STROBE) reporting guidelines.

TRIAD included 268 participants who had no cognitive impairment (134 individuals [50%]), MCI (63 [23.5%]), AD dementia (46 [17.2%]), and non-AD dementia (24 [9.0%]). The WRAP study^26^ included data on 323 participants, predominantly without cognitive impairment at the first plasma sample collection (no impairment, 309 [95.6%]; MCI, 12 [3.7%]; AD dementia, 2 [0.6%]). The SPIN cohort^27^ included 195 participants: controls without cognitive impairment (82 [42.1%]), individuals with MCI due to AD (72 [36.7%]), and individuals with AD dementia (41 [21.0%]). Diagnosis was based on internationally recognized clinical criteria, and control participants had normal cognitive scores on standard neuropsychological evaluations. A subset of patients with longitudinal follow-up consisted of 392 participants from TRIAD and WRAP defined by PET biomarkers (eTable 1 in Supplement 1). These included participants classified into 3 groups: amyloid- and tau-negative (A−T−; n = 297), amyloid-positive and tau-negative (A+T−; n = 66), and amyloid-positive and tau-positive (A+T+; n = 29). In WRAP, the median number of samples collected per patient was 3 over a mean (SD) of 5.22 (1.41) years. In TRIAD, median samples per patient was 2, collected over a mean of 1.90 (0.61) years.

### Imaging, CSF, and Plasma Biomarkers

Detailed imaging methods for TRIAD, WRAP, and SPIN are found in the eMethods in Supplement 1. In TRIAD, Aβ and tau PET were determined by [^18^F]-AZD4694^28^ and [^18^F]-MK6240,^29^ respectively. In WRAP, PET measures were determined by [^11^C]-PiB^30^ and [^18^F]-MK6240.^31,32^ In SPIN, Aβ PET was determined by [^18^F]-florbetapir or [^18^F]-flutemetamol in a smaller subset of participants, with CSF Aβ42/40 used to define A status for most participants as described below. Tau PET was not available for the SPIN cohort, and T was defined by CSF p-tau181. Aβ-PET positivity was standardized across cohorts as a centiloid value greater than 24 (standardized uptake value ratio [SUVR] >1.55 for [^18^F]-AZD4694^33^ or distribution volume ratio >1.2 for [^11^C]-PiB). Tau positivity with [^18^F]-MK6240 was defined as meta-temporal region of interest SUVR greater than 1.24 for TRIAD^34^ and SUVR greater than 1.3 in WRAP.

CSF sample collection procedures were similar across cohorts and are described in the eMethods in Supplement 1. In TRIAD and SPIN, Lumipulse G1200 or G600II was used to quantify CSF Aβ42, Aβ40, and p-tau181.^35,36^ Additionally, CSF p-tau217 was quantified by an in-house single-molecule array (Simoa) developed at the University of Gothenburg.^33^ A novel Simoa for CSF p-tau205 was measured in TRIAD only. For WRAP, CSF Aβ42, Aβ40, and p-tau181 were measured using the Roche NeuroToolKit.^37^

Plasma samples from TRIAD, WRAP, and SPIN were analyzed at the Department of Psychiatry and Neurochemistry, University of Gothenburg. Plasma Aβ42/40, glial fibrillary acidic protein (GFAP), and neurofilament light chain (NfL) were quantified using the commercial Neurology 4-plex E kit (103670; Quanterix). Plasma p-tau231 and p-tau181 were analyzed using in-house Simoa assays developed at the University of Gothenburg,^18,38^ except in WRAP where plasma p-tau181 was quantified by the commercial Advantage kit version 2.1 (104111; Quanterix).^24^

### Novel p-Tau217 Assay

The commercial ALZpath pTau217 assay for p-tau217 uses a proprietary monoclonal p-tau217 specific capture antibody, an N-terminal detector antibody, and a peptide calibrator. It has been validated as a fit-for-purpose assay^38^ with a limit of detection of 0.0052 to 0.0074 pg/mL, a functional lower limit of quantification of 0.06 pg/mL, and a dynamic range of 0.007 to 30 pg/mL. The spike recovery for the endogenous analyte was 80%, and intrarun and interrun precision was 0.5% to 13% and 9.2% to 15.7%, respectively. Here, the assay demonstrated good repeatability (4%-8.7%) and intermediate precision (3.5%-10.7%) as shown in eTable 2 in Supplement 1.

### Statistical Analysis

Between-group comparisons were conducted using linear models, adjusting for age and sex. Determining Aβ-PET and tau-PET positivity and other outcomes was done using receiver operating characteristics AUC and compared with those of other established biomarkers with the DeLong test. Correlations were always evaluated using Spearman ρ. A binary reference point for Aβ-PET positivity was derived based on the Youden index.

Alternatively, a 3-range strategy comprised a lower reference point to rule out AD (95% sensitivity) and a higher reference point to rule in AD (95% specificity). In both strategies, we evaluated the concordance of a negative p-tau217 result with Aβ-PET negativity (negative percent agreement), and the concordance of a positive plasma p-tau217 with Aβ-PET positivity (positive percent agreement), as well as the overall percent agreement. In the latter strategy, individuals with p-tau217 levels between the reference point were classified as intermediate risk and would constitute the population referred to confirmatory testing.^39^

We evaluated the longitudinal trajectories of plasma p-tau217 in participants with no cognitive impairment and those with MCI according to their amyloid (A) and tau (T) status. We used linear mixed-effects models with plasma p-tau217 as the response variable, including as predictors time (since first plasma collection), AT status, age at first plasma collection, years of education, sex, and cognitive status at first visit, as well as an interaction between AT status and time. The model contained random intercepts and random slopes for each participant, and time was modeled as a continuous variable. Post hoc pairwise contrasts were conducted to compare the slopes for group × time interactions.

All analyses were performed using R version 4.2.2 (R Project for Statistical Computing), with a 2-sided α of .05. No adjustments for multiple comparisons were performed.^40^ Reported results include 95% confidence intervals when applicable.

## Results

### Participant Characteristics

A total of 786 participants (mean [SD] age, 66.3 [9.7] years; 504 females [64.1%], 282 males [35.9%]) were included in the study. The TRIAD subsample included 268 participants (69.4 [7.8] years; 167 females [62.3%], 101 males [37.7%]). The WRAP cohort included 323 participants (65.3 [6.9] years; 217 females [67.2%], 106 males [32.8%]), predominantly without cognitive impairment. The SPIN cohort included 195 participants (63.5 [13.8] years; 120 females [61.5%], 75 males [38.5%]). All participants had confirmatory amyloid status (TRIAD and WRAP: Aβ PET; SPIN: CSF Aβ42/Aβ40), and the majority (716 [91.1%]) also had information on tau status (TRIAD and WRAP: tau PET; SPIN: CSF p-tau181), as described in Table 1 alongside demographic and clinical information for all cross-sectional analyses. eTable 2 in Supplement 1 describes the TRIAD and WRAP longitudinal subsets.

**Table 1.** Cross-Sectional Demographic Data for the WRAP, TRIAD, and SPIN Cohorts

Characteristic	Mean (SD)		
	WRAP (n = 323)	TRIAD (n = 268)	SPIN (n = 195)
Age, y	65.3 (6.91)	69.4 (7.90)	63.5 (13.8)
Sex, No. (%)			
Female	217 (67.2)	167 (62.3)	120 (61.5)
Male	106 (32.8)	101 (37.7)	75 (38.5)
*APOE* ε4 carriers, No. (%)	121 (37.5)	96 (35.8)	81 (41.5)
MMSE score	29.2 (1.23)	27.0 (4.72)	26.4 (4.19)
Baseline clinical diagnosis, No. (%)			
No cognitive impairment	309 (95.7)	134 (50.0)	82 (42.1)
Cognitive impairment	14 (4.3)	134 (50.0)	113 (57.9)
Years of education	16.1 (2.62)	15.0 (3.57)	13.4 (5.12)
AT status, No. (%)			
A−T−	209 (78.9)	146 (55.3)	75 (41.2)
A+T−	38 (14.3)	65 (24.6)	6 (3.3)
A+T+	18 (6.8)	53 (20.1)	101 (55.5)
A−T+	1 (0.3)	2 (0.7)	2 (1.0)
Missing data^a^	57 (17.6)	2 (0.7)	11 (5.6)
Plasma p-tau217, pg/mL	0.466 (0.362)	0.636 (0.648)	0.977 (0.766)

Abbreviations: Aβ, amyloid β; A+, amyloid-positive; A−, amyloid-negative; CSF, cerebrospinal fluid; MMSE, Mini-Mental State Examination; PET, positron emission tomography; p-tau, phosphorylated tau; SPIN, Sant Pau Initiative on Neurodegeneration; T+, tau-positive; T−, tau-negative; TRIAD, Translational Biomarkers in Aging and Dementia; WRAP, Wisconsin Registry for Alzheimer’s Prevention.

^a^ In the WRAP and TRIAD cohorts, AT status was defined with amyloid and tau PET. In WRAP, all participants had available Aβ-PET data (100%), while tau PET was not available for 57 participants (17.6%). In TRIAD, all participants had available Aβ-PET data (100%), and tau PET was not available for 2 participants (0.7%). In SPIN, all participants had data for amyloid status, which was determined with CSF Aβ42/Aβ40 in 159 (71.5%) participants or with Aβ PET in 36 (18.5%) participants. In SPIN, tau status was defined with CSF p-tau181 and was not available for 11 participants (5.6%).

### p-Tau217 Levels by Amyloid and Tau Status

**Figure 1.**
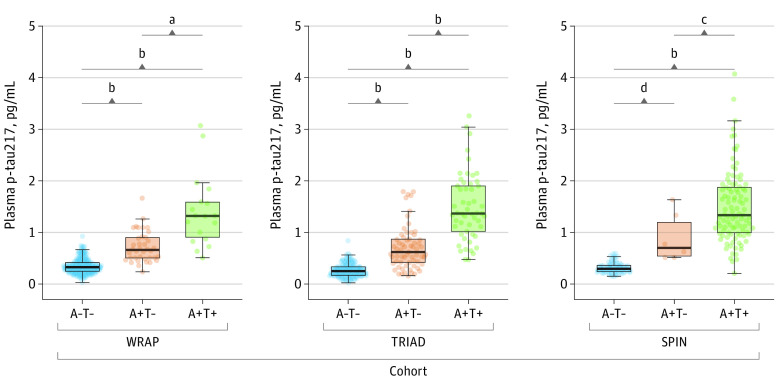
Plasma Phosphorylated Tau 217 (p-Tau217) Levels According to Amyloid β (A) and Tau (T) Profiles Boxplots show the distribution of p-tau217 concentrations by AT profile for the Wisconsin Registry for Alzheimer’s Prevention (WRAP), Translational Biomarkers in Aging and Dementia (TRIAD), and Sant Pau Initiative on Neurodegeneration (SPIN) cohorts. For WRAP and TRIAD, Aβ and tau were indexed by positron emission tomography. In SPIN, A was indexed by cerebrospinal fluid (CSF) Aβ42/40 and T by CSF p-tau181. All comparison *P* values obtained from pairwise contrasts from linear models adjusted for age and sex were less than .001, whereas in the SPIN cohort, 2 comparisons showed *P* < .05. The horizontal line inside each box indicates the median, the outer bounds of boxes represent lower and upper quartiles, and whiskers extend to the 5th and 95th IQRs; circles indicate observed data points. ^a^*P* = .001. ^b^*P* < .001. ^c^A+T− vs A+T+: *P* = .03. ^d^A−T− vs A+T−: *P* = .02.

When stratified by AT status, regardless of clinical diagnosis, plasma p-tau217 significantly increased in a stepwise manner in all cohorts (Figure 1), with highest levels in the A+T+ group. Mean p-tau217 concentrations for A−T− (mean [SD] TRIAD, 0.26 [0.13] pg/mL; WRAP, 0.35 [0.15] pg/mL; SPIN, 0.32 [0.11] pg/mL), A+T− (TRIAD, 0.75 [0.63] pg/mL; WRAP, 0.72 [0.30] pg/mL; SPIN, 0.91 [0.47] pg/mL), and A+T+ (TRIAD, 1.48 [0.65] pg/mL; WRAP, 1.41 [0.70] pg/mL; SPIN, 1.50 [0.70] pg/mL) were remarkably similar across all 3 cohorts. This was also observed when stratifying by amyloid status alone (A−, TRIAD, 0.28 [0.21] pg/mL; WRAP, 0.35 [0.14] pg/mL; SPIN, 0.38 [0.29] pg/mL; and A+, TRIAD, 1.08 [0.72] pg/mL, WRAP, 0.94 [0.54] pg/mL, SPIN, 1.43 [0.70] pg/mL) (eFigure 1 in Supplement 1).

### Accuracy in Discriminating Abnormal Aβ and Tau Pathologies

**Figure 2.**
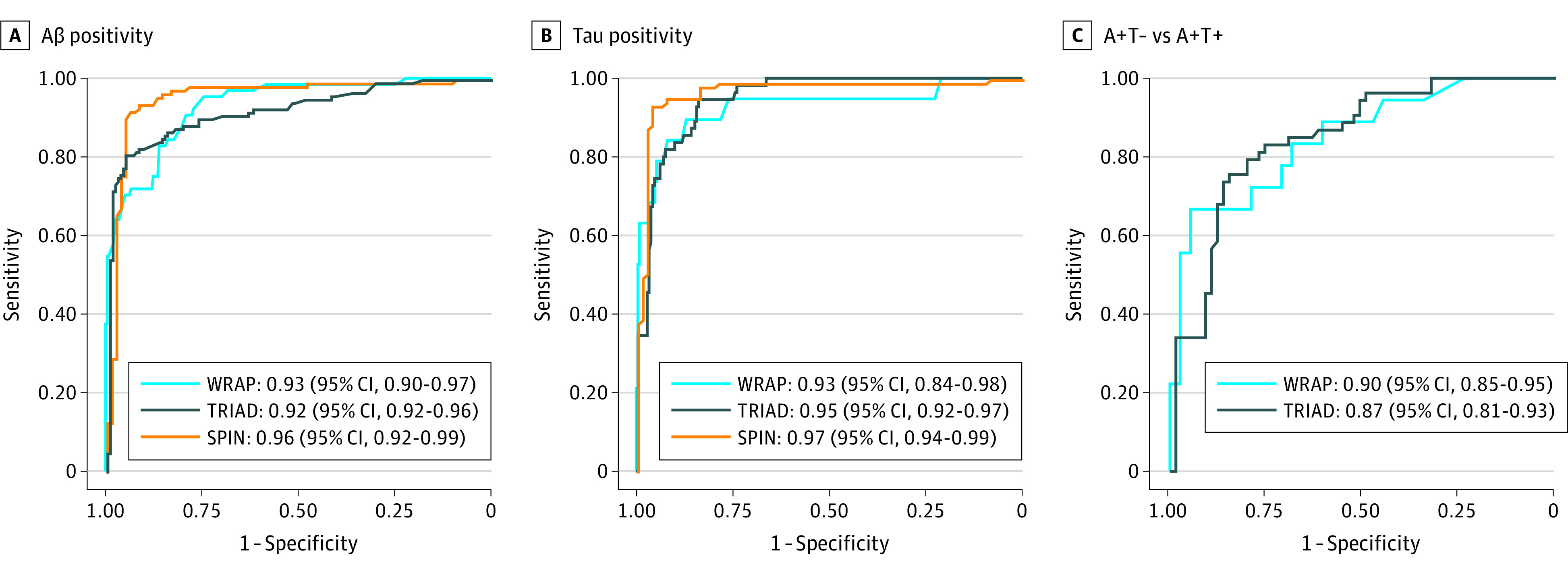
Accuracy of the Phosphorylated Tau 217 (p-Tau217) Immunoassay in Detecting Amyloid β (Aβ) Positivity and Tau (T) Positivity and Discriminating A+T− From A+T+ Individuals Receiver operating characteristics (ROC) curves for p-tau217 in detecting Aβ positivity and tau positivity and to differentiating Aβ-positive and tau-positive individuals (A+T+) from Aβ-positive and tau-negative (A+T−). For each ROC curve, the area under the curve is reported alongside 95% CI. For WRAP and TRIAD, Aβ and tau were indexed by positron emission tomography. In SPIN, A was indexed by cerebrospinal fluid (CSF) Aβ42/40 and T by CSF p-tau181.

Plasma p-tau217 demonstrated high accuracy in predicting abnormal Aβ-PET signal (centiloid >24) in TRIAD (AUC, 0.92; 95% CI, 0.92-0.96) and WRAP (AUC, 0.93; 95% CI, 0.90-0.97) (Figure 2A). In SPIN, p-tau217 also had high accuracy in predicting abnormal CSF Aβ42/40 (AUC, 0.96; 95% CI, 0.92-0.99) (Figure 2A). There was equally high accuracy when Aβ-PET status was determined by visual read (eFigure 2 in Supplement 1). Further, p-tau217 sustained high accuracy when Aβ-PET status was defined by differing centiloid values (eg, centiloid >12 and centiloid >37) in TRIAD and WRAP participants (eFigure 3 in Supplement 1).

Plasma p-tau217 also exhibited high accuracy for predicting abnormal tau in TRIAD (AUC, 0.95; 95% CI, 0.92-0.97) and WRAP (AUC, 0.93; 95% CI, 0.84-0.98) (Figure 2B). In SPIN, p-tau217 had high accuracy for abnormal CSF p-tau181 (AUC, 0.97; 95% CI, 0.94-0.99). Promisingly, p-tau217 could identify abnormal tau PET signal among amyloid-positive participants (A+T− vs A+T+) in TRIAD (AUC, 0.87; 95% CI, 0.81-0.93) and WRAP (AUC, 0.90; 95% CI, 0.85-0.95) (Figure 2C). Moreover, we observed a gradual increase of plasma p-tau217 across tau-PET–defined Braak stages in TRIAD (eFigure 4 and eTable 3 in Supplement 1).

### Comparing p-Tau217 With Imaging and CSF Biomarkers in Identifying AD Pathology

Next, we compared the performance of plasma p-tau217 to CSF and imaging modalities for predicting abnormal Aβ PET and tau PET. This analysis included the maximum number of participants within each biomarker modality. In WRAP, in determining abnormal Aβ PET, plasma p-tau217 outperformed hippocampal atrophy (AUC, 0.52; 95% CI, 0.44-0.60; *P* < .001), tau PET (AUC, 0.72; 95% CI, 0.64-0.80; *P* < .001), and CSF p-tau181 (AUC, 0.75; 95% CI, 0.66-0.84; *P* < .001) but did not differ significantly from CSF Aβ42/40 or CSF p-tau181/Aβ42 (eFigure 5A in Supplement 1). Similar findings were observed in TRIAD, where plasma p-tau217 outperformed hippocampal atrophy (AUC, 0.70; 95% CI, 0.63-0.76; *P* < .001) and tau PET (AUC, 0.86; 95% CI, 0.82-0.91; *P* = .05) for detecting abnormal Aβ pathology but did not significantly differ from various CSF biomarkers (eFigure 5B in Supplement 1). In SPIN, plasma p-tau217 outperformed hippocampal volume (AUC, 0.89; 95% CI, 0.83-0.95; *P* = .04) and was comparable with CSF biomarkers (eFigure 5C in Supplement 1).

In predicting abnormal tau-PET burden (eFigure 5D-E in Supplement 1), plasma p-tau217 significantly outperformed hippocampal volume (WRAP AUC, 0.65; 95% CI, 0.50-0.81; *P* = .01; TRIAD AUC, 0.83; 95% CI, 0.76-0.89; *P* = .01; SPIN AUC, 0.91; 95% CI, 0.86-0.96, *P* = .049). Plasma p-tau217 significantly outperformed CSF p-tau181 in WRAP (AUC, 0.69; 95% CI, 0.66-0.84; *P* = .02) but not TRIAD. Plasma p-tau217 outperformed Aβ PET in TRIAD (AUC, 0.90; 95% CI, 0.86-0.95; *P* = .04), while in WRAP they were comparable (AUC, 0.96; 95% CI, 0.93-0.99; *P* = .35). Plasma p-tau217 showed comparable performance with other measures, except for CSF p-tau217 in SPIN.

Additionally, we conducted comparisons in subsets only including participants with all modalities (WRAP: n = 131; TRIAD: n = 106; SPIN: n = 41), finding no marked differences (eFigure 6 in Supplement 1). Plasma p-tau217 also discriminated A+T+ from A+T− individuals comparably with CSF and imaging biomarkers (eFigure 7 in Supplement 1).

### Comparing p-Tau217 With Other Plasma Biomarkers

Plasma p-tau217 alone or p-tau217 plus demographic variables (age, sex, and *APOE* status) outperformed all other plasma biomarkers (p-tau181, p-tau231, Aβ42/40, GFAP, and NfL), and their optimal combinations, for predicting both amyloid and tau status in all cohorts (eTables 4 and 5 and eFigure 7 in Supplement 1). A minimal improvement in model metrics of goodness-of-fit (Akaike information criterion) was observed in p-tau217 plus demographic data but not in discriminatory performance. The correlations of plasma p-tau217 with Aβ PET, tau PET, and CSF p-tau217 are shown in eFigures 9 and 10 in Supplement 1.

### Reference Ranges for Plasma p-Tau217 With Abnormal Aβ and Tau Pathologies

We first derived a binary reference point for Aβ positivity using the Youden index, derived in WRAP (>0.42 pg/mL) (Table 2 and eFigure 11 in Supplement 1). This reference point was cross-validated in TRIAD (Aβ positivity based on PET) and SPIN (Aβ positivity based on CSF Aβ42/40). We next applied a 3-range approach,^41^ creating lower (95% sensitivity, <0.4 pg/mL) and upper (95% specificity, >0.63 pg/mL) reference points in WRAP (Table 2 and eFigure 11 in Supplement 1). This approach improved the positive percent agreement (TRIAD: 97.7%; SPIN: 95.3%) while maintaining a similar negative percent agreement. The “intermediate” zone (p-tau217 levels 0.4-0.63 pg/mL), which could in practice be referred to confirmatory testing with CSF or PET, was largest in WRAP (22.9%), as expected because of lower Aβ-positivity prevalence, and smaller in TRIAD (15.8%) and SPIN (13.0%). A binary reference point for tau positivity is demonstrated in eTable 6 in Supplement 1.

**Table 2.** Binary Reference and Three-Range Reference for Aβ Positivity^a^

Characteristic	Binary reference for Aβ positivity: plasma p-tau217 >0.42 pg/mL			Characteristic	Three-range reference for Aβ positivity: plasma p-tau217 positive >0.63 pg/mL, plasma p-tau217 negative <0.40 pg/mL		
	WRAP	TRIAD	SPIN		WRAP	TRIAD	SPIN
No. of participants	323	268	195	No. of participants	323	268	195
Aβ-positive, No. (%)	64 (19.8)	120 (44.8)	110 (56.4)	Aβ-positive, No. (%)	64 (19.8)	120 (44.8)	110 (56.4)
Plasma p-tau217 status positive, No. (%)	127 (39.3)	124 (46.3)	127 (65.1)	Plasma p-tau217 positive, No. (%)	58 (18.0)	86 (32.1)	106 (54.4)
				Plasma p-tau217 intermediate, No. (%)	74 (22.9)	43 (16.0)	24 (12.3)
				Plasma p-tau217 negative, No. (%)	191 (59.1)	139 (51.9)	65 (33.3)
Sensitivity, %	95.3	85.0	98.2	Sensitivity of lower reference point, %	95.3	86.7	98.2
Specificity, %	74.5	85.1	77.6	Specificity of upper reference point, %	94.9	98.6	94.1
PPA, %	48.0	82.3	85.0	PPA, upper reference point, %	77.6	97.7	95.3
NPA, %	98.5	87.5	97.1	NPA, lower reference point, %	98.4	88.5	96.9
OPA, %	78.6	85.1	89.2	OPA for p-tau217 positive and negative, %	93.6	92.0	95.9

Abbreviations: Aβ, amyloid β; NPA, negative percent agreement; OPA, overall percent agreement; PPA, positive percent agreement; p-tau217, phosphorylated tau 217; SPIN, Sant Pau Initiative on Neurodegeneration; TRIAD, Translational Biomarkers in Aging and Dementia; WRAP, Wisconsin Registry for Alzheimer’s Prevention.

^a^ The table shows key metrics for the evaluation of a binary and 3-range reference point for Aβ positivity. The binary reference point was based in the Youden index derived in the WRAP cohort and cross-validated in the TRIAD and SPIN cohorts. Three-range reference points for Aβ positivity were derived in WRAP based on 95% sensitivity (lower reference point) and 95% specificity (upper reference point) and cross-validated in TRIAD and SPIN. The OPA for p-tau217 negative and positive indicates the combined NPA of those below the lower reference point and the PPA for those above the upper reference point, not accounting for the intermediate zone. In WRAP and TRIAD, Aβ positivity was determined with Aβ positron emission tomography, whereas in SPIN, Aβ positivity was determined with cerebrospinal fluid Aβ42/Aβ40.

### Longitudinal Changes in Plasma p-Tau217 Levels

**Figure 3.**
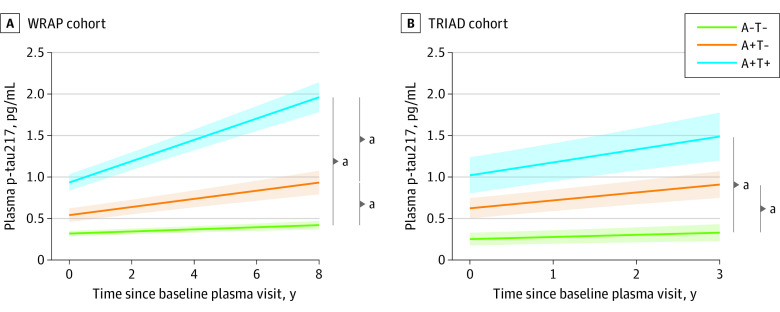
Longitudinal Trajectories of Plasma Phosphorylated Tau 217 (p-Tau217) Values According to Amyloid β (A) and Tau (T) Status by Positron Emission Tomography (PET) Trajectory plots indicate the mean longitudinal trajectories (solid line) of plasma p-tau217 and associated 95% CIs (shading), estimated with linear mixed-effects models. Trajectories are stratified based on PET-defined amyloid and tau groups (A−T−, A+T−, A+T+), modeled with an interaction term between AT status and time. Models included random slopes and intercepts for each participant and were adjusted for years of education, sex, and cognitive status at first visit. *P* values represent post hoc pairwise comparing the slopes for group × time interactions. ^a^*P* < .001.

In up to 8 years of longitudinal sampling in WRAP (mean [SD], 5.22 [1.41] years), the A+T+ group demonstrated a significantly higher annual increase rate in plasma p-tau217 levels compared with the A−T− group (β estimate, 0.12; 95% CI, 0.10-0.13; *P* < .001). The A+T− group also demonstrated a significantly higher annual rate of change in plasma p-tau217 compared with A−T− (β estimate, 0.04; 95% CI, 0.02-0.05; *P* < .001). Slope comparisons showed the A+T+ group to have a significantly higher rate compared with the A+T− group (β estimate, 0.08; 95% CI, 0.06-0.09; *P* < .001) (Figure 3A). In TRIAD, similar results were observed, even with a shorter follow-up (mean [SD], 1.90 [0.61] years) (Figure 3B).

## Discussion

In 3 independent cohorts, this study presents the performance of a commercially available plasma assay targeting p-tau217. Our findings demonstrate high accuracy in identifying abnormal Aβ and tau pathologies, comparable with CSF measures and superior to brain atrophy assessments. A 3-range approach demonstrated high negative and positive concordance with Aβ status, with approximately 20% of individuals in an intermediate zone that would require confirmatory CSF or PET, as previously proposed.^41^ Longitudinally, this assay exhibited increases solely in individuals with Aβ pathology at baseline, and those with both elevated Aβ and tau pathologies demonstrated a greater rate of annual increase.

Plasma biomarkers have emerged as important tools for AD evaluation. Their specificity to underlying pathology offers great potential for rapid screening, reducing the dependence on advanced confirmatory tests. A clinical AD diagnosis often lacks sensitivity and specificity, resulting in many individuals with MCI (40%-60%) or dementia (20%-30%) who exhibit typical AD symptoms lacking Aβ pathology.^1^ In primary care, it is estimated that more than 50% of patients with cognitive impairment remain undiagnosed or incorrectly diagnosed because of the lack of accessible and cost-effective tools.^1^ Thus, blood biomarkers are set to revolutionize clinical care by providing objective biomarker-based information. As anti-Aβ trials move toward targeting a preclinical population with lower prevalence of Aβ abnormalities,^42^ a cost-effective screening strategy becomes paramount. In previous studies, targeting p-tau217 in blood has yielded the best results as a diagnostic and prognostic tool that tracks longitudinal change.

There has been limited access to immunoassays targeting p-tau217 for broader evaluation. This study evaluates a commercially available assay for p-tau217 that exhibits similar advantageous features to those previously reported. Consistent with Palmqvist et al,^19^ this assay outperformed magnetic resonance imaging and showed comparable performance with CSF biomarkers in detecting Aβ PET positivity and tau PET positivity.^43^ Further, significant superiority to other plasma p-tau epitopes, Aβ42/40, NfL, and GFAP and their optimal combinations was shown. When combined with *APOE* status and age, only modest improvements in diagnostic accuracy were observed, whereas other plasma biomarkers relied more heavily on these variables for their performance. Notably, the assay demonstrated high accuracy in identifying tau pathology within Aβ-positive individuals. This is particularly important as antiamyloid therapies may be less effective in patients with advanced tau pathology.^44,45^ Our findings suggest that p-tau217 has the potential to identify elevated tau-PET uptake and promising utility in early AD trials. Our study did not define elevated tau in the same manner as the TRAILBLAZER trials but warrants further studies applying p-tau217 to intermediate-tau trial inclusion designs.^45^

Integrating blood biomarkers into diagnostic workflows remains challenging despite their promise. Therefore, this study also aimed to establish reference points based on abnormal Aβ pathology. The study evaluated a 3-range approach as recommended by Alzheimer’s Association guidelines^39^ and recently proposed by Brum et al,^41^ which suggests confirmatory testing for patients with uncertain plasma p-tau217 results. Evaluating this approach using a commercial immunoassay showed high negative and positive predictive accuracy at screening, indicating only 12% to 23% of individuals warranted advanced testing, depending on the clinical stage. However, we acknowledge that the cohorts used in this study may not fully represent real-world clinical settings. Importantly, the reported negative and positive predictive accuracy of these reference ranges can vary based on the prevalence of the outcome in the target population. Lower positive percent agreements are expected in settings with lower prevalence,^46^ as observed in the preclinical WRAP cohort compared with the higher prevalence seen in TRIAD and SPIN cohorts. Therefore, future studies should prospectively evaluate plasma p-tau217 reference points in memory clinic populations with wider diversity to ensure optimized implementation, accounting for higher rates of important comorbidities.^47^

### Limitations

This study is not without limitations. First, one-third of our participants were classified as cognitively impaired, and this may limit our generalizability to the symptomatic stages of the disease but highlights promise for future preclinical recruitment. In addition, our results cannot be generalized to all individuals without detailed examination in cohorts with a larger representation of diverse ethnic populations. We acknowledge that CSF p-tau181, utilized as a T marker in SPIN, is not interchangeable with other methods that more accurately reflect neurofibrillary tangle pathology.^48^

## Conclusions

This study highlights the effectiveness of a commercially available plasma p-tau217 assay in identifying AD pathology. Our findings demonstrate the substantial reduction of confirmatory testing, by approximately 80%, by implementing a 3-range approach for Aβ positivity based on plasma p-tau217. These results emphasize the important role of plasma p-tau217 as an initial screening tool in the management of cognitive impairment by underlining those who may benefit from antiamyloid immunotherapies.

## Notes

Supplement 1.
eMethods
**eFigure 1.** Plasma ALZpath pTau217 levels according to amyloid status **eFigure 2.** Plasma ALZpath pTau217 levels according to amyloid status defined based on PET visual reads **eFigure 3.** Plasma ALZpath pTau217 levels according to amyloid status defined by Centiloid values >12 and >37 **eFigure 4.** Plasma ALZpath pTau217 levels according to Braak stages in TRIAD **eFigure 5.** Plasma ALZpath pTau217 demonstrates similar or superior diagnostic accuracy for Aβ and tau pathologies compared to established CSF and PET biomarkers **eFigure 6.** Plasma ALZpath pTau217 also demonstrates similar or superior diagnostic accuracy for Aβ and tau pathologies compared to established CSF and PET biomarkers when evaluated in full biomarker availability subset **eFigure 7.** Plasma pTau217 accuracy for discriminating tau pathology status among amyloid-positive individuals is superior to that of established biomarkers **eFigure 8.** Plasma pTau217 demonstrates higher accuracy for Aβ and tau positivity compared to other plasma biomarkers and their combinations **eFigure 9.** Correlations of ALZpath pTau217 with Aβ and tau PET **eFigure 10.** Correlations of ALZpath pTau217 with CSF pTau217 **eFigure 11.** Binary and three-range ALZpath pTau217 reference ranges for Aβ-positivity **eTable 1.** Demographics of longitudinal cohort **eTable 2.** Intermediate precision and repeatability of the ALZpath pTau217 assay **eTable 3.** ALZpath pTau217 levels by Braak stage **eTable 4.** Receiver operating characteristics curves of plasma biomarkers to determine Aβ positivity **eTable 5.** Receiver operating characteristics curves of plasma biomarkers to determine tau positivity **eTable 6.** Binary reference for tau-positivity

Supplement 2. Data sharing statement
