# Supplementary material for: Diagnostic Accuracy of a Plasma Phosphorylated Tau 217 Immunoassay for Alzheimer Disease Pathology
Source: JAMA Neurol. 2024 Jan 22;81(3):255–63. doi: 10.1001/jamaneurol.2023.5319 (PMC10804282; doi:10.1001/jamaneurol.2023.5319)
Supplement: Supplement 1. — eMethods eFigure 1. Plasma ALZpath pTau217 levels according to amyloid status eFigure 2. Plasma ALZpath pTau217 levels according to amyloid status defined based on PET visual reads eFigure 3. Plasma ALZpath pTau217 levels according to amyloid status defined by Centiloid values >12 and >37 eFigure 4. Plasma ALZpath pTau217 levels according to Braak stages in TRIAD eFigure 5. Plasma ALZpath pTau217 demonstrates similar or superior diagnostic accuracy for Aβ and tau pathologies compared to established CSF and PET biomarkers eFigure 6. Plasma ALZpath pTau217 also demonstrates similar or superior diagnostic accuracy for Aβ and tau pathologies compared to established CSF and PET biomarkers when evaluated in full biomarker availability subset eFigure 7. Plasma pTau217 accuracy for discriminating tau pathology status among amyloid-positive individuals is superior to that of established biomarkers eFigure 8. Plasma pTau217 demonstrates higher accuracy for Aβ and tau positivity compared to other plasma biomarkers and their combinations eFigure 9. Correlations of ALZpath pTau217 with Aβ and tau PET eFigure 10. Correlations of ALZpath pTau217 with CSF pTau217 eFigure 11. Binary and three-range ALZpath pTau217 reference ranges for Aβ-positivity eTable 1. Demographics of longitudinal cohort eTable 2. Intermediate precision and repeatability of the ALZpath pTau217 assay eTable 3. ALZpath pTau217 levels by Braak stage eTable 4. Receiver operating characteristics curves of plasma biomarkers to determine Aβ positivity eTable 5. Receiver operating characteristics curves of plasma biomarkers to determine tau positivity eTable 6. Binary reference for tau-positivity [file jamaneurol-e235319-s001.pdf]

Supplemental Online Content

Ashton NJ, Brum WS, Di Molfetta G, et al. Diagnostic accuracy of a plasma phosphorylated tau 217 immunoassay for Alzheimer disease pathology. *JAMA Neurol*. Published online January 22, 2024. doi:10.1001/jamaneurol.2023.5319

eMethods
eFigure 1. Plasma ALZpath pTau217 levels according to amyloid status
eFigure 2. Plasma ALZpath pTau217 levels according to amyloid status defined based on PET visual reads
eFigure 3. Plasma ALZpath pTau217 levels according to amyloid status defined by Centiloid values >12 and >37
eFigure 4. Plasma ALZpath pTau217 levels according to Braak stages in TRIAD
eFigure 5. Plasma ALZpath pTau217 demonstrates similar or superior diagnostic accuracy for Aβ and tau pathologies compared to established CSF and PET biomarkers
eFigure 6. Plasma ALZpath pTau217 also demonstrates similar or superior diagnostic accuracy for Aβ and tau pathologies compared to established CSF and PET biomarkers when evaluated in full biomarker availability subset
eFigure 7. Plasma pTau217 accuracy for discriminating tau pathology status among amyloid-positive individuals is superior to that of established biomarkers
eFigure 8. Plasma pTau217 demonstrates higher accuracy for Aβ and tau positivity compared to other plasma biomarkers and their combinations
eFigure 9. Correlations of ALZpath pTau217 with Aβ and tau PET
eFigure 10. Correlations of ALZpath pTau217 with CSF pTau217
eFigure 11. Binary and three-range ALZpath pTau217 reference ranges for Aβ-positivity
eTable 1. Demographics of longitudinal cohort
eTable 2. Intermediate precision and repeatability of the ALZpath pTau217 assay
eTable 3. ALZpath pTau217 levels by Braak stage
eTable 4. Receiver operating characteristics curves of plasma biomarkers to determine Aβ positivity
eTable 5. Receiver operating characteristics curves of plasma biomarkers to determine tau positivity
eTable 6. Binary reference for tau-positivity
eReferences

This supplemental material has been provided by the authors to give readers additional information about their work.

© 2024 Ashton NJ et al. *JAMA Neurology.*

eMethods

The Translational Biomarkers in Aging and Dementia (TRIAD)

TRAID is an observational and longitudinal biomarker study approved by the Montreal Neurological Institute PET working committee and the Douglas Mental Health University Institute Research Ethics Board. Written informed consent was obtained for all participants. TRIAD participants are followed yearly with detailed clinical and neuropsychological assessments, as well as with collection of biofluids (blood, urine, saliva, and CSF) and acquisition of multiple imaging biomarkers. This study included cross-sectional data on 268 participants from TRIAD with multimodal imaging Aβ PET [^{18}F]-AZD4694, tau PET [^{18}F]-MK6240 and magnetic resonance imaging (MRI). A subset of participants with imaging had corresponding CSF biomarkers (Aβ42/40, pTau181, pTau205, ptau217, pTau231). The included participants were classified as cognitively unimpaired (CU, *n(%)* = 134(50%)), mild cognitive impairment (MCI, *n(%)* = 63(23.5%)), AD (*n(%)* = 46(17.2%)) and non-AD dementia (*n(%)* = 24(9.0%)). CU individuals had no objective cognitive impairment and a Clinical Dementia Rating (CDR) score of 0. Individuals with MCI had subjective and/or objective cognitive impairment and a CDR score of 0.5. Individuals with dementia had a CDR score of 1 or 2. Structural MRI data was acquired on a 3T Siemens Magnetom to obtain a high-resolution T1-weighted image of the entire brain. T1-weighted anatomical images were segmented using the SPM12 segmentation tool and non-linearly registered to the ADNI template using DARTEL, as previously reported [1]. Brain atrophy was estimated using hippocampal volume, which measurements were estimated using FreeSurfer and were adjusted for total intracranial volume (ICV), as previously described [2]. ICV adjustment was performed based on data from CU participants at baseline. T1-weighted anatomical images were also employed for coregistration purposes to PET images. A Siemens High Resolution Research Tomograph (HRRT) was used for PET imaging acquisitions, which occurred ±80 days from the CSF collection date (median = 53vdays). For Aβ PET, images were acquired 40–70 minutes post-injection of [^{18}F]-AZD4694 and scans were reconstructed using the ordered subset expectation maximization (OSEM) algorithm on a 4-dimensional volume with 3 frames (3x600s) [3]. For tau PET, [^{18}F]-MK6240 scans were acquired 90–110 minutes post-injection and the OSEM algorithm was also used for reconstruction on a 4D volume with 4 frames (4x300s). Additional pre-processing corrections were performed as described elsewhere [4]. PET images were meninges and skull stripped, linearly and non-linearly registered to the ADNI template space and then spatially smoothed to achieve a final resolution of 8 mm FWHM [5]. The inferior cerebellum and whole cerebellum gray matter were used as the reference regions for [^{18}F]-MK6240 and [^{18}F]-AZD4694, respectively. Global Aβ PET used averaged SUVR of the precuneus, cingulate, inferior parietal, medial prefrontal, lateral temporal, and orbitofrontal cortices and a positivity value of 1.55 [6], corresponding to 24 Centiloids [7]. Aβ positivity was also visually defined by two neurologists blinded to clinical diagnosis. Tau PET SUVR was globally estimated from a composite area including the meta-

© 2024 Ashton NJ et al. *JAMA Neurology.*

ROI region. Tau positivity was defined 1.24 as previously described [8] (mean + 2 standard deviations (SD) higher than the mean meta-ROI region of the young (<26 years of age) participants).

CSF samples were collected by syringe and transferred to polypropylene tubes for centrifugation at 20 °C, 2200g for 10 minutes. Samples were then distributed into 1 millilitre aliquots in polypropylene vials (Fisher Scientific Inc. Catalog # 3741-WP1D-BR) and permanently stored at -80 °C pending biochemical analyses at the Department of Neurochemistry, University of Gothenburg. CSF pTau181 and Aβ42/40 were quantified by the LUMIPULSE G1200 as previously described [6]. CSF pTau217 [9] and pTau205 (Lantero-Rodriguez et al., unpublished) were quantified by in-house Simoa assay developed at the University of Gothenburg. All plasma analysed for TRIAD was performed at the Department of Psychiatry and Neurochemistry, University of Gothenburg. Plasma Aβ42/40, GFAP and NfL were quantified by the commercial Neurology 4-plex E (#103670, Quanterix). Plasma pTau181 and pTau231 were analysed by in-house Simoa assays developed at the University of Gothenburg [10, 11]. Plasma pTau217 was quantified by the ALZpath Simoa assay as described in the manuscript methods at the Department of Psychiatry and Neurochemistry, University of Gothenburg between 1st December 2022 and 22nd December 2022.

Wisconsin Registry for Alzheimer's Prevention (WRAP)

The WRAP study and the measures collected for this project were approved by the University of Wisconsin-Madison institutional review board and written informed consent was obtained for all participants. WRAP [12] is an observational longitudinal observational cohort that collects biofluid, cognitive and clinical data at approximate biennial visits. An expanding subset of participants undergo amyloid PET [^{11}C]-PiB, tau PET [^{18}F]-MK6240, MRI and/or CSF collection procedures. Participants who had provided suitable plasma (with EDTA anticoagulant which began in 2011) and had undergone at least one lumbar puncture or at least one [^{11}C]-PiB were eligible to be included. The majority of those with [^{11}C]-PiB data also underwent tau PET with [^{18}F]-MK6240. This study included cross-sectional data on 323 WRAP participants, and mostly CU (CU, *n(%)* = 309(95.6%) at their first available plasma sample collection with no clinically significant cognitive impairment based on a consensus panel review. Some individuals at first available plasma sample draw had cognitive impairment MCI, *n(%)* = 12(3.7%); dementia, *n(%)* = 2(0.6%)) using standard diagnostic criteria. All participants with PET also had acquired 3T T1-weighted images for co-registration purposes. PET imaging was acquired on a Siemens HR+. For Aβ PET, images were acquired dynamically 0–70 minutes post-injection of PiB as described previously from which mean cortical distribution volume ratios (DVR) was derived using Logan graphical analysis with the cerebellum gray matter as the reference region [13]. For tau PET, [^{18}F]-MK6240 scans were acquired 70-90 minutes post-injection and meta-temporal ROI standard uptake value ratios (SUVR) were derived as described elsewhere [14, 15]. PET images were spatially registered with the MNI template and the AAL3 (in the case of PiB) or Harvard Oxford atlas (for tau PET) standard regions were extracted. For WRAP the global cortical Aβ PET utilized the averaged DVR of the

© 2024 Ashton NJ et al. *JAMA Neurology.*

precuneus, anterior and posterior cingulate, inferior parietal, medial prefrontal, lateral temporal, and orbitofrontal cortices and positivity was defined as a DVR of 1.19 or greater which corresponded to a Centiloid >21.7) as described elsewhere [16]. For the purposes of this study, an additional Aβ PET Centiloid of 24 was used for purposes of comparison to the TRIAD cohort. Tau PET SUVR was assessed in the temporal meta-ROI, a commonly used summary measure of tau PET [17] that encompasses the entorhinal cortex, amygdala, parahippocampal gyrus, fusiform gyrus, inferior and middle temporal gyrus. Tau positivity was defined as [^{18}F]-MK6240 temporal meta-ROI SUVR > 1.30 (unpublished) which was 2.5 standard deviations (SD) higher than the mean of middle-aged (< 60), amyloid negative participants.

CSF samples were collected by syringe and transferred to polypropylene tubes for centrifugation at 20 °C, 2200 g for 10 min. Samples were then distributed into 0.5mL aliquots in polypropylene vials and permanently stored at -80 °C pending analyses. CSF biomarkers (Aβ42, Aβ40, pTau181 among others) using the Roche NeuroToolKit [18] were measured at the Department of Psychiatry and Neurochemistry, University of Gothenburg. All plasma analysed for this study was performed at the Department of Psychiatry and Neurochemistry, University of Gothenburg, and identical to TRIAD, with exception of plasma pTau181, which was quantified by the commercial pTau-181 Advantage V2.1 Simoa (#104111, Quanterix). Plasma pTau217 was quantified by the ALZpath Simoa assay as described in the manuscript methods at the Department of Psychiatry and Neurochemistry, University of Gothenburg between 5th Janurary 2023 and 3rd February 2023.

Sant Pau Initiative on Neurodegeneration (SPIN)

The SPIN cohort is a comprehensive observational platform for studying neurodegenerative diseases that uses multiple types of biomarkers and takes an integrative approach [19]. Individuals who participate in SPIN agree to donate biofluid (blood and CSF) and undergo detailed neurological and neuropsychological evaluations. A subset of participants also receives a 3T brain MRI scan and additional functional or imaging studies such as a video-polysomnogram, [^{18}F]-fluorodeoxyglucose PET, amyloid PET, or Tau PET. Participants are followed for at least 4 years, with additional samples and imaging studies taken every other year. All procedures in the study were approved by the ethics committee of Hospital Sant Pau, and all participants or their legally authorized representatives provided written informed consent in accordance with the Declaration of Helsinki. For cross-sectional analysis, we included 195 participants with biomarker confirmation (CSF or PET). We included individuals with mild cognitive impairment due to (MCI-AD, *n(%)* = 72(36.7%)), AD dementia (AD, *n(%)* = 41(21.0%)) and and cognitively unimpaired controls (CU, *n(%)* = 82(42.1%)). Diagnosis was based on internationally recognized clinical criteria, and control participants had normal cognitive scores on standard neuropsychological evaluations. A subset of participants had structural 3T-MRI and/or amyloid PET ([^{18}F]-florbetapir or [^{18}F]-flutemetamol, n = 36). The Computational Anatomy Toolbox (CAT12, http://dbm.neuro.uni-jena.de/cat), which is a tool for the SPM12 software, was used to pre-process the structural T1 sequence of the MRI and to extract hippocampal volumes [20]. PET images were

© 2024 Ashton NJ et al. *JAMA Neurology.*

co-registered to the corresponding MRI of everyone. The amyloid PET images were intensity-scaled using the cerebellum region. All resulting PET images were projected to the middle point of the cortical ribbon, inspected visually for potential errors, and smoothed with a 10mm kernel. We normalized the amyloid PET images to the MNI space and calculated the standardized uptake value ratio (SUVR) using the target amyloid region and whole cerebellum regions of the GAAIN website (www.gaain.org). The SUVR values were then converted to the Centiloid scale [21].

CSF samples were collected in 10 mL polypropylene tubes (Sarstedt, #62.610.018), which were then taken to the Sant Pau Memory Unit laboratory. Within 2 hours, samples were centrifuged, aliquoted, and stored at -80°C. CSF levels of core AD biomarkers (Aβ42, Aβ40, and pTau181) were measured in the Lumipulse fully-automated platform using commercially available kits (Fujirebio Europe, Ghent, Belgium), as previously described [21].

Blood samples were collected in 10 ml EDTA-2K tubes and then centrifuged for 10 minutes at 4°C. All plasma analysed for this study was performed at the Department of Psychiatry and Neurochemistry, University of Gothenburg, and identical to TRIAD. Plasma pTau217 was quantified by the ALZpath Simoa assay as described in the manuscript methods at the Department of Psychiatry and Neurochemistry, University of Gothenburg between 20th March 2023 and 30rd March 2023.

© 2024 Ashton NJ et al. *JAMA Neurology.*

eFigure 1. Plasma ALZpath pTau217 levels according to amyloid status.

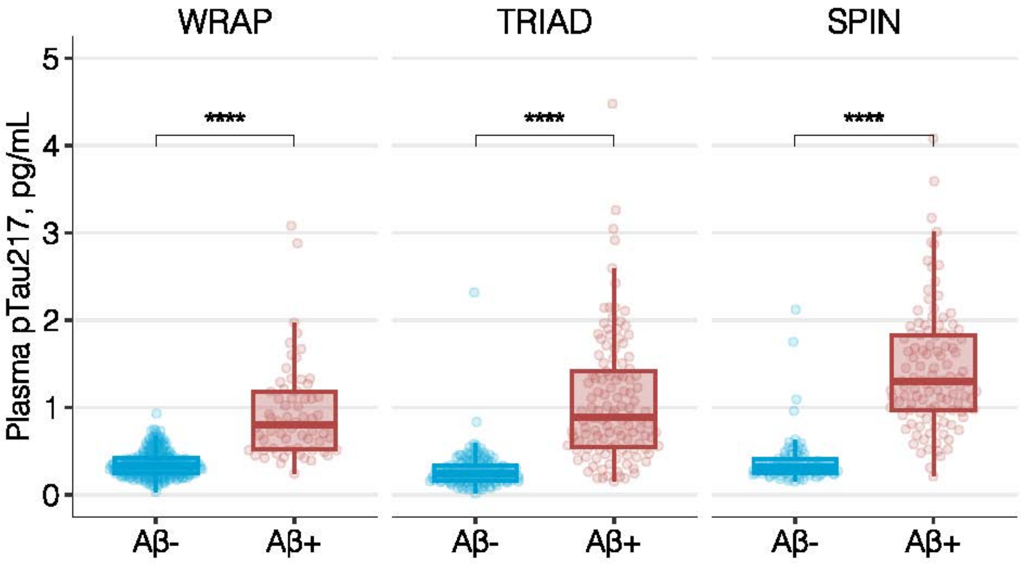

Boxplots show the distribution of ALZpath pTau217 value by Aβ profile for the WRAP, TRIAD, and SPIN cohorts. For WRAP and TRIAD, Aβ ("A") was indexed by PET. In SPIN, Aβ ("A") was indexed by CSF Aβ42/40. P-values were obtained from pairwise contrasts performed on linear models controlling for age and sex, and all were <0.0001 (****), with Tukey multiplicity adjustment.

© 2024 Ashton NJ et al. *JAMA Neurology.*

eFigure 2. Plasma ALZpath pTau217 levels according to amyloid status defined based on PET visual reads.

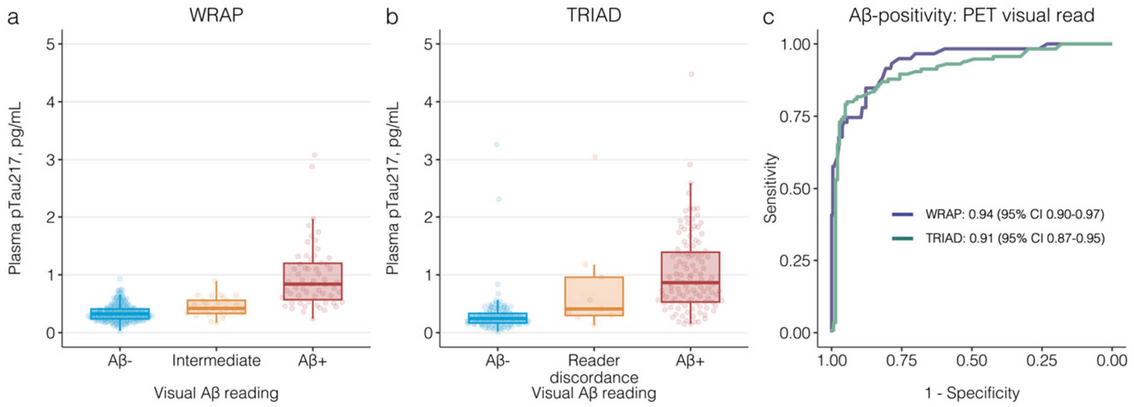

Boxplots show the distribution of ALZpath pTau217 value by visual Aβ PET reading for WRAP (A) and TRIAD (B). Receiver operating characteristics (ROC) curves for ALZpath pTau217 for Aβ positivity by visual read in both WRAP and TRIAD (C). For each ROC curve, the area under the curve (AUC) is reported alongside 95% confidence intervals (CI).

© 2024 Ashton NJ et al. *JAMA Neurology.*

eFigure 3. Plasma ALZpath pTau217 levels according to amyloid status defined based Centiloid >12 and Centiloid >37.

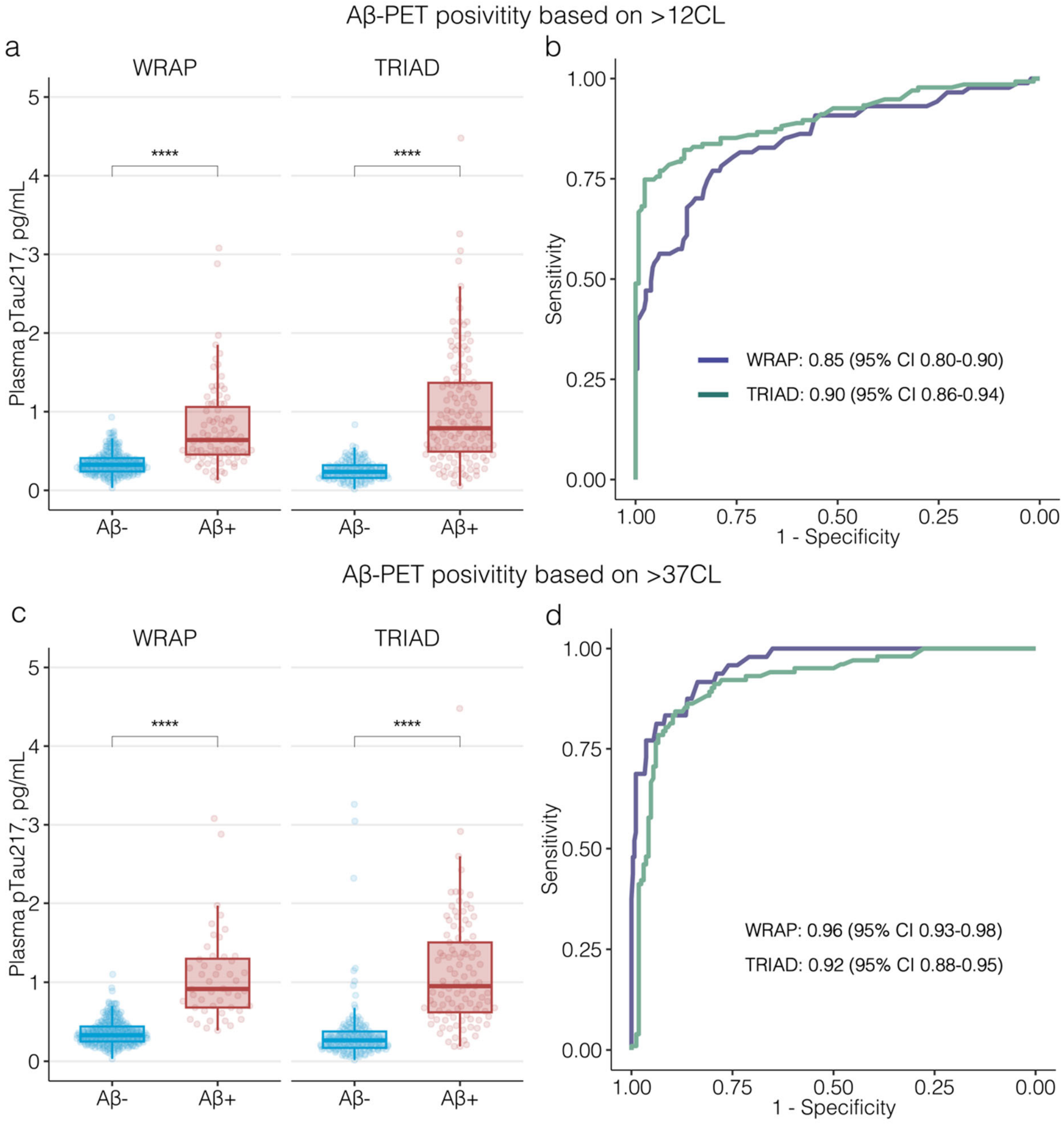

Boxplots show the distribution of ALZpath pTau217 levels and receiver operating characteristics (ROC) show its discriminatory ability for Aβ PET positivity defined based on a cutoff of 12CL (A-B) and of 37CL (C-D) for WRAP and TRIAD. CL=centiloid.

© 2024 Ashton NJ et al. *JAMA Neurology.*

eFigure 4 – Plasma ALZpath pTau217 levels according to Braak stages in TRIAD.

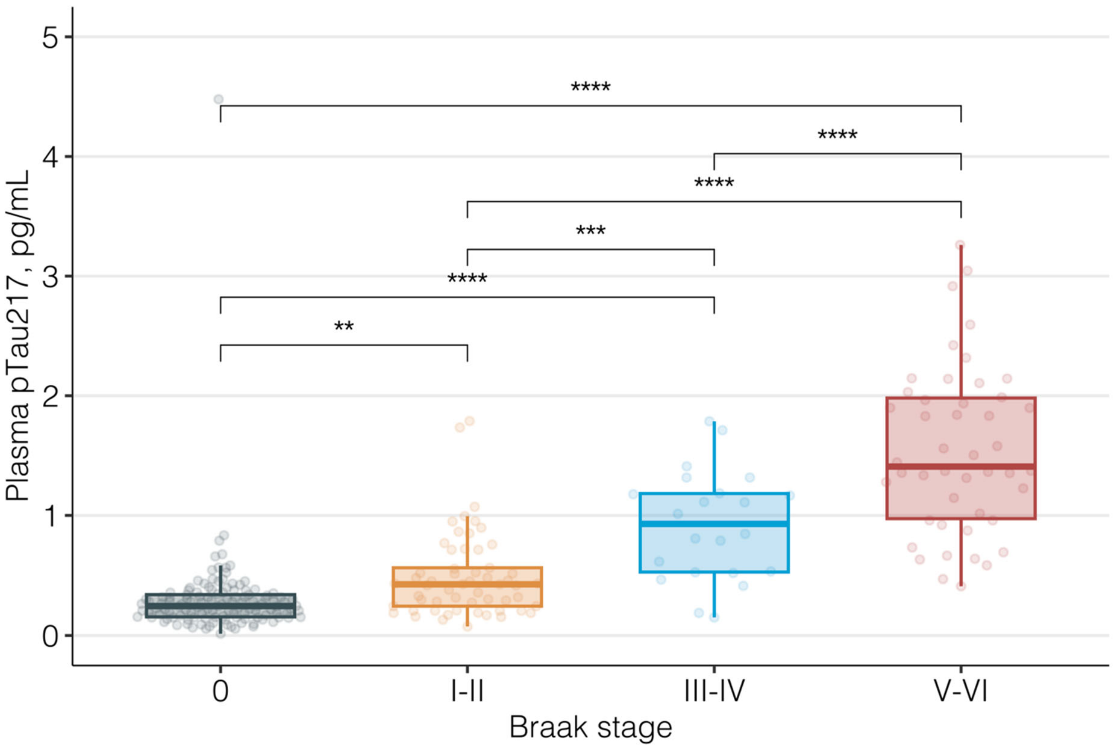

Boxplots show the distribution of ALZpath pTau217 levels by Braak stage determined by tau PET profile for the TRIAD cohort. P-values were obtained from pairwise contrasts performed on a linear model adjusted for age and sex, with Tukey multiplicity adjustment.

$P_{0vsI\text{-}II}$=0.22 (ns); $P_{I\text{-}IIvsIII\text{-}IV}$=0.0042 (***); all others P<0.0001 (****).

© 2024 Ashton NJ et al. *JAMA Neurology.*

eFigure 5. Plasma ALZpath pTau217 demonstrates similar or superior diagnostic accuracy for Aβ and tau pathologies compared to established CSF and PET biomarkers.

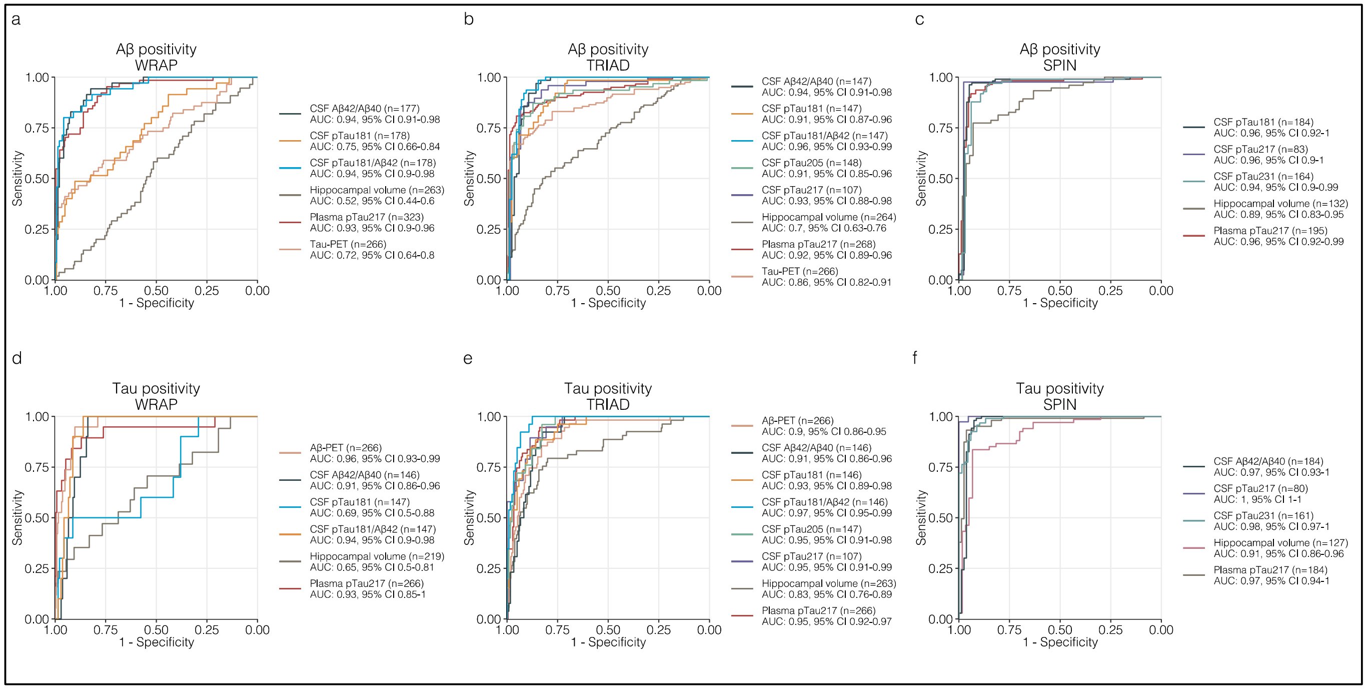

Receiver operating characteristics curves for detecting Aβ (A-C) and tau-positivity (D-F) for ALZpath pTau217, CSF and imaging biomarkers. Each panel corresponds to accuracies for the same outcome at each cohort (WRAP: A, D; TRIAD: B, E; SPIN: C, F). Solid lines represent the ROC curves for each biomarker, with colors corresponding to a specific biomarker across cohorts, as indicated in the figure legend at each panel. The legend indicates the area under the curve (AUC) for each biomarker, alongside 95% confidence intervals (CI). The maximum number of observations with complete data for each biomarker and outcome were used, and the specific number for each biomarker is also detailed in the figure legend. This approach, in which no data is excluded, yielded similar results to AUC comparisons within a reduced subset including the same subjects with complete data for all biomarkers, reported in the Supplement. For WRAP and TRIAD, Aβ ("A") and tau ("T") were indexed by PET. In SPIN, "A" was indexed by CSF Aβ42/40 and "T" by CSF pTau181.

© 2024 Ashton NJ et al. *JAMA Neurology.*

eFigure 6. Plasma ALZpath pTau217 demonstrates similar or superior diagnostic accuracy for Aβ and tau pathologies compared to established CSF and PET biomarkers when evaluated in full biomarker availability subset.

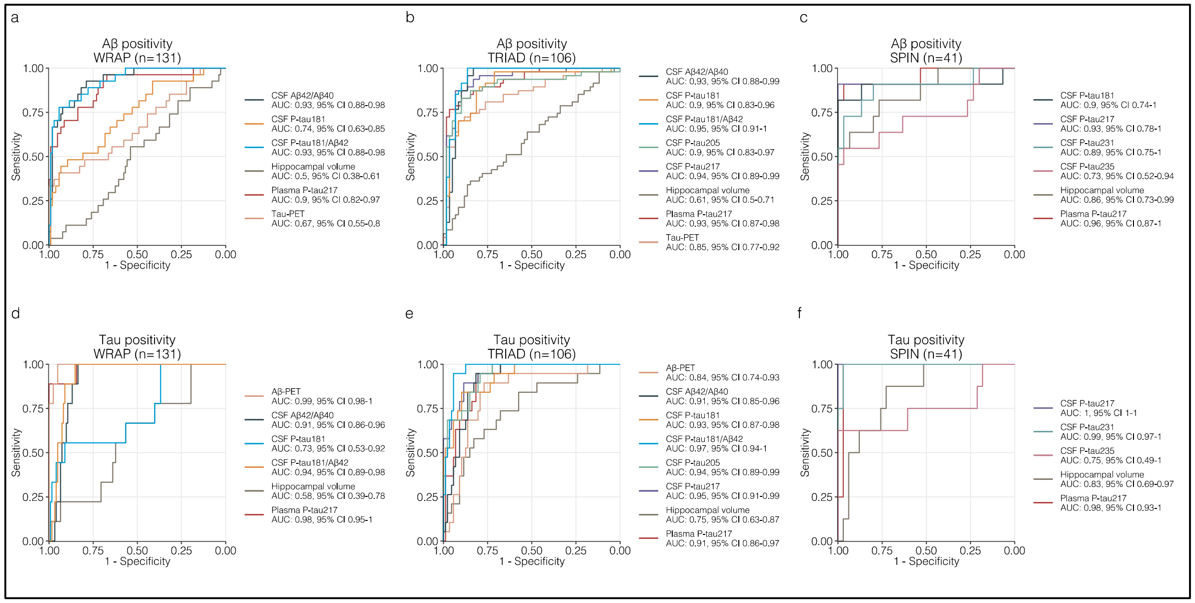

Receiver operating characteristics curves for detecting Aβ (A-C) and tau-positivity (D-F) for ALZpath pTau217, CSF and imaging biomarkers. Each panel corresponds to accuracies for the same outcome at each cohort (WRAP: A, D; TRIAD: B, E; SPIN: C, F). For WRAP and TRIAD, Aβ ("A") and tau ("T") were indexed by PET. In SPIN, "A" was indexed by CSF Aβ42/40 and "T" by pTau181. Solid lines represent the ROC curves for each biomarker, with colors corresponding to a specific biomarker across cohorts, as indicated in the figure legend at each panel. The legend indicates the area under the curve (AUC) for each biomarker, alongside 95% confidence intervals (CI). As indicated in the title of each panel, this analysis was conducted in a same subset of participants who had available data for all biomarkers included in the comparison. This approach led to similar results to the one presented in the main text, in which no data is excluded and the maximum number of observations per biomarker was used.

© 2024 Ashton NJ et al. *JAMA Neurology.*

eFigure 7. Plasma pTau217 accuracy for discriminating tau pathology status among amyloid-positive individuals is superior to that of established biomarkers.

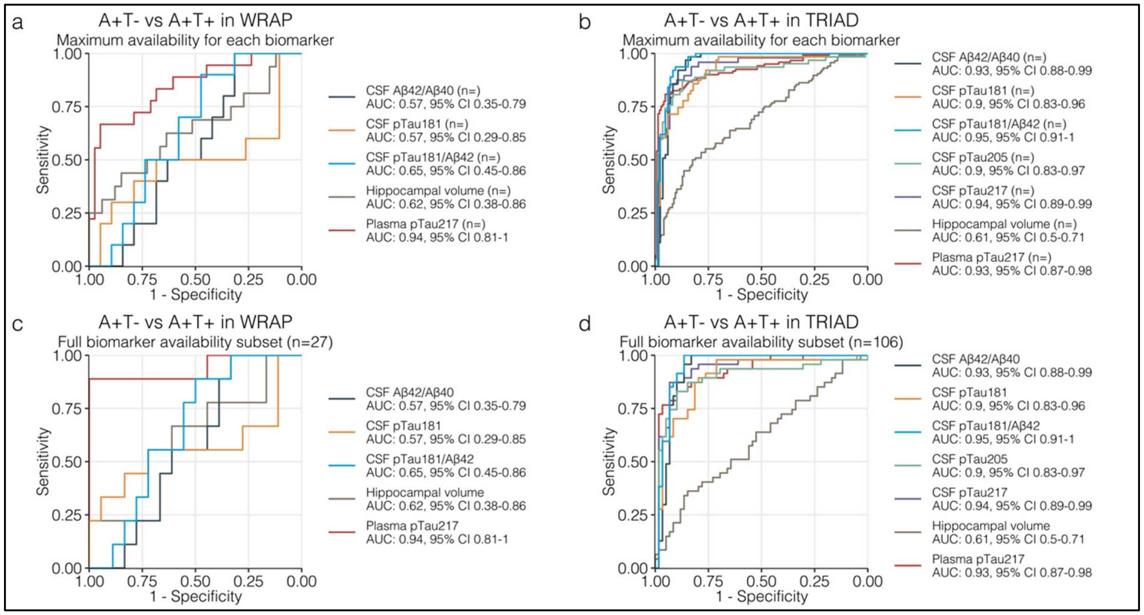

Receiver operating characteristics (ROC) curves for ALZpath pTau217 for discriminating A+T+ from A+T- in WRAP (A, C) and TRIAD (B, D), both modalities defined by PET. In (A) and (B), the analyses were performed based on the maximum number of available observations for each biomarker-outcome combination, while in (C) and (D) analyses were conducted in reduced subsets with those participants who had available data for all biomarkers. The legend indicates the area under the curve (AUC) for each biomarker, alongside 95% confidence intervals (CI).

© 2024 Ashton NJ et al. *JAMA Neurology.*

eFigure 8. Plasma pTau217 demonstrates higher accuracy for Aβ and tau positivity compared to other plasma biomarkers and their combinations.

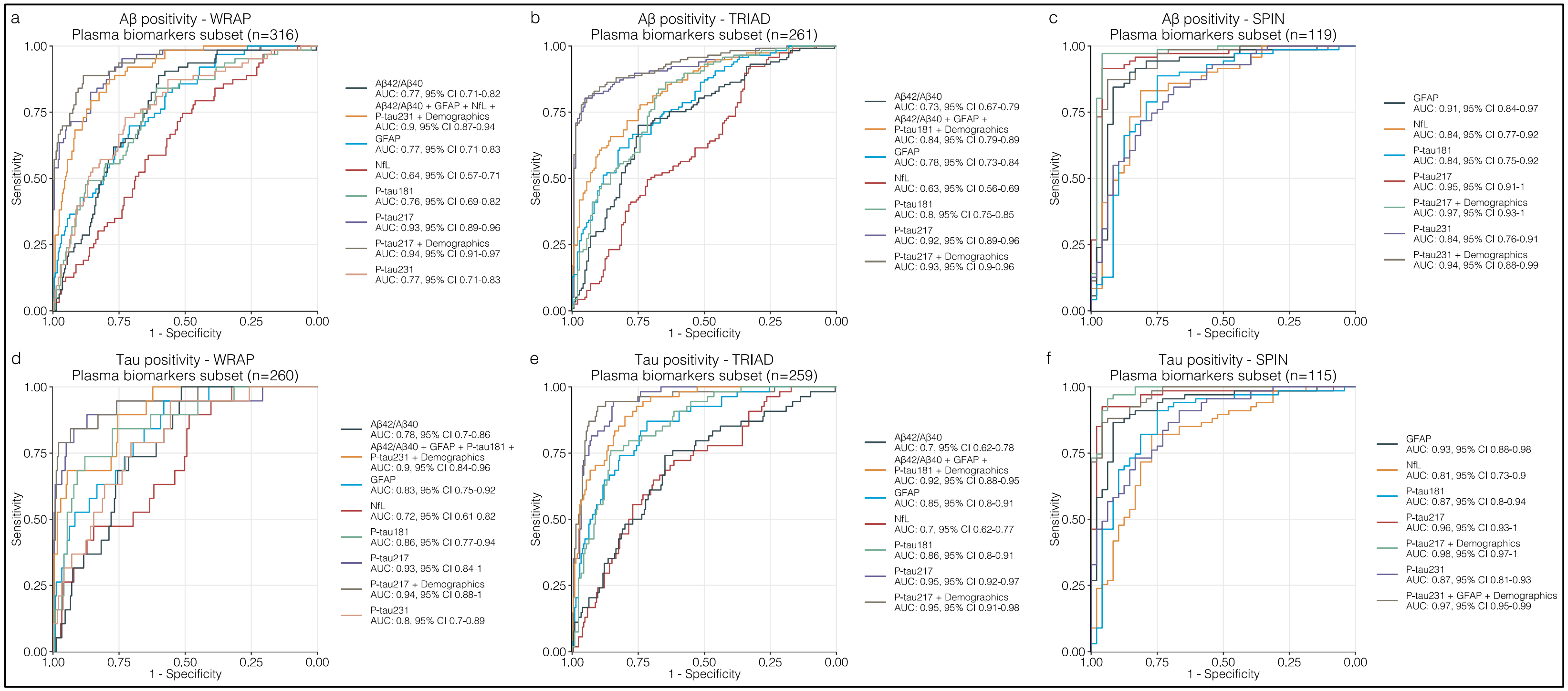

Receiver operating characteristics (ROC) curves for plasma biomarkers and plasma biomarker combinations to detect Aβ positivity (A-C) and tau positivity (D-F). The legend indicates the area under the curve (AUC) for each plasma biomarker or plasma biomarker combination, alongside 95% confidence intervals (CI). For WRAP and TRIAD, Aβ (“A”) and tau (“T”) were indexed by PET. In SPIN, “A” was indexed by CSF Aβ42/40 and “T” by pTau181. Biomarker combinations were evaluated based on model metrics shown in eTables 4-5. “Demographics” refers to the addition of age, sex and *APOE* status.

© 2024 Ashton NJ et al. *JAMA Neurology*.

eFigure 9. Correlations of ALZpath pTau217 with Aβ and tau PET.

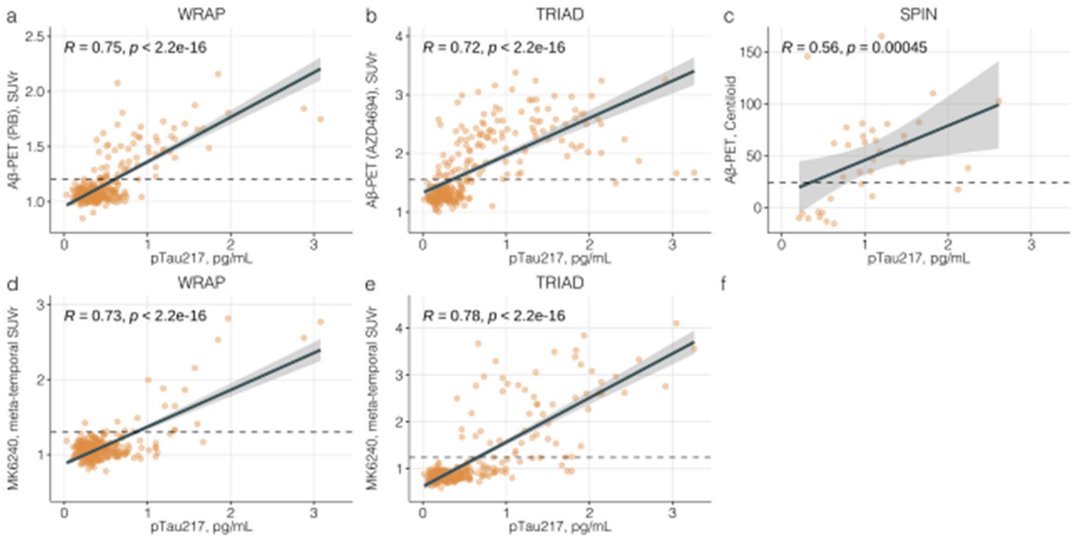

Correlations of ALZpath pTau217 with Aβ PET in WRAP (A), TRIAD (B) and SPIN (C), and between pTau217 and tau-PET in WRAP (D) and TRIAD (E). Dots correspond to individual datapoints. Correlation coefficients correspond to Spearman's rho. The Aβ- or tau-PET ligand is indicated in the y-axis, except for SPIN, in which Aβ-PET is represented in the centiloid scale since patients underwent [^{18}F]-flutemetamol or [^{18}F]-florbetapir.

© 2024 Ashton NJ et al. *JAMA Neurology.*

eFigure 10. Correlations of ALZpath pTau217 with CSF pTau217.

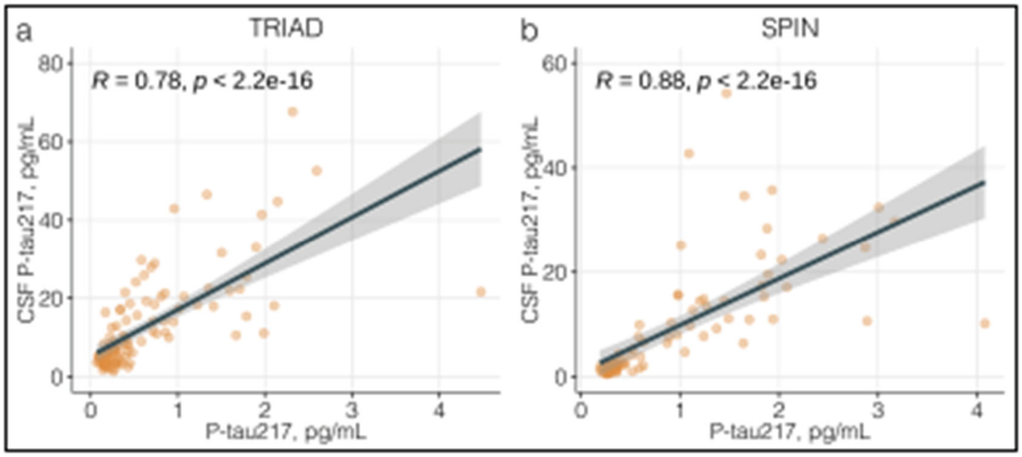

Correlations of ALZpath pTau217 with CSF pTau217, TRIAD (A) and SPIN (B). Correlation coefficients correspond to Spearman's rho. CSF pTau217 measures were not available in WRAP.

© 2024 Ashton NJ et al. *JAMA Neurology.*

eFigure 11. Binary and three-range ALZpath pTau217 reference ranges for Aβ-positivity.

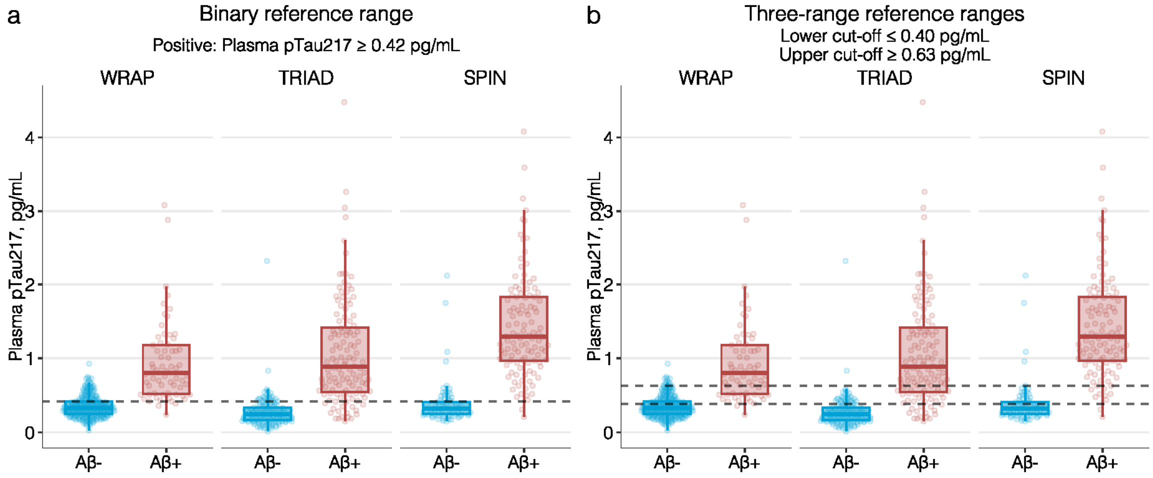

Boxplots show the distribution of plasma ALZpath pTau217 according to Aβ status (Aβ-negative: blue; Aβ-positive: red) for each of the three cohorts alongside reference ranges. (A) The dashed line represents a binary cut-off (>0.42 pg/mL) for Aβ-positivity derived based on the conventionally used Youden index. (B) The upper dashed line represents an upper cut-point (>0.63 pg/mL) for considering a pTau217 reading as positive, derived with 95% specificity for Aβ-positivity. The lower dashed line represents a lower cut-point (<0.40 pg/mL), below which plasma pTau217 would be considered negative, derived with 95% sensitivity for Aβ-positivity. For WRAP and TRIAD, Aβ ("A") and tau ("T") were indexed by PET. In SPIN, "A" was indexed by CSF Aβ42/40 and "T" by CSF pTau181.

© 2024 Ashton NJ et al. *JAMA Neurology.*

eTable 1 – Demographics of longitudinal cohort.

	WRAP			TRIAD		
	A-T-<br>(N=217)	A+T-<br>(N=26)	A+T+<br>(N=17)	A-T-<br>(N=80)	A+T-<br>(N=40)	A+T+<br>(N=12)
Age, years, mean (SD)	61.5 (6.54)	64.0 (5.26)	65.0 (5.71)	70.5 (5.94)	73.8 (5.04)	70.1 (5.58)
Female, n (%)	144 (66.4%)	15 (57.7%)	14 (82.4%)	50 (62.5%)	25 (62.5%)	8 (66.7%)
APOE ε4 carriers, n (%)	75 (34.6%)	17 (65.4%)	13 (76.5%)	21 (26.3%)	14 (35.0%)	8 (66.7%)
Baseline MMSE score, mean (SD)	29.4 (0.887)	29.0 (1.22)	27.5 (3.10)	29.0 (1.27)	28.5 (1.39)	27.3 (2.00)
Baseline clinical diagnosis						
CU, n (%)	212 (97.7%)	26 (100%)	17 (100%)	70 (87.5%)	21 (52.5%)	0 (0%)
CI, n (%)	5 (2.3%)	0 (0%)	0 (0%)	10 (12.5%)	19 (47.5%)	12 (100%)
Years of education, mean (SD)	16.2 (2.76)	16.8 (2.15)	15.7 (2.23)	15.5 (3.83)	15.1 (3.16)	14.7 (3.70)
Baseline plasma pTau217, pg/mL, mean (SD)	0.333 (0.142)	0.553 (0.339)	0.895 (0.395)	0.260 (0.141)	0.808 (0.744)	1.07 (0.447)
Mean (SD) years of follow-up	5.28 (1.38)	5.06 (1.47)	4.76 (1.71)	1.95 (0.618)	1.87 (0.620)	1.68 (0.546)
Median (IQR) number of samples	3 (3-4)	3 (3-3)	3 (2-3)	2 (2-3)	2 (2-3)	2 (2-2.25)

Data are mean (SD) or n (%). In both cohorts, AT status was defined with amyloid and tau-PET. Abbreviations: SD, standard deviation; TRIAD, Translational Biomarkers in Aging and Dementia; WRAP, Wisconsin Registry for Alzheimer's Prevention; MMSE, mini-metal state examination; CU, cognitively unimpaired; CI, cognitively impaired; A-T-, amyloid-negative and tau-negative; AT-, amyloid-positive and tau-negative; A-T+, amyloid-positive and tau-positive. For WRAP and TRIAD, Aβ ("A") and tau ("T") were indexed by PET.

© 2024 Ashton NJ et al. *JAMA Neurology.*

eTable 2 – Intermediate precision and repeatability of the ALZpath pTau217 assay.

Quality Controls (range, pg/mL)	Repeatability (% CV_r); Intermediate precision (%CV_{Rw})		
	TRIAD	WRAP	SPIN
IQC-1 (0.5–0.7)	8.1; 8.2	10.6; 13.6	4.5; 8.1
IQC-2 (1.6–2.0)	6.9; 9.6	10.3; 17.9	4.0; 7.6
IQC-3 (2.3)	6.6; 9.7	8.2;13.5	5.3; 7.1
EQC-1 (0.77–0.88)	1.5; 8.8	1.1;3.6	1.7; 7.1
EQC-2 (0.14- 0.2)	3.5; 11.0	1.9;6.9	2.2; 7.5

Precision of plasma ALZpath pTau217 assay in all cohorts. IQC samples are human plasma samples from the University of Gothenburg. EQC samples are plasma samples provided by ALZpath commercial assay.

eTable 3 – ALZpath pTau217 levels by Braak stage in the TRIAD cohort.

Braak Stage	n	ALZpath pTau217, pg/mL (SD)
0	124	0.30 (0.41)
I-II	57	0.49 (0.35)
III-IV	22	0.92 (0.46)
V-VI	46	1.55 (0.70)

The table indicates the mean (SD) concentrations of plasma ALZpath pTau217 in the TRIAD cohort, According to Braak stages defined with [^{18}F]MK-6240.

© 2024 Ashton NJ et al. *JAMA Neurology.*

eTable 4 – Receiver operating characteristics curves of plasma biomarker to determine Aβ-status.

Cohort	Biomarker	AUC	CI Lower	CI Upper	AIC
WRAP	pTau217	0.931	0.897	0.965	161
	pTau231	0.774	0.711	0.838	274
	Aβ42/Aβ40	0.770	0.713	0.827	284
	GFAP	0.766	0.704	0.828	271
	pTau181	0.765	0.701	0.829	289
	NfL	0.650	0.581	0.720	312
	Demographics + pTau217	0.940	0.905	0.974	148
	Demographics + Aβ42/Aβ40 + GFAP + NfL + pTau231	0.903	0.866	0.940	209
	Demographics + Aβ42/Aβ40 + GFAP + NfL + pTau181+ pTau231	0.904	0.867	0.941	211
	Demographics + Aβ42/Aβ40 + GFAP + pTau231	0.893	0.853	0.933	215
	Demographics + Aβ42/Aβ40 + GFAP + pTau181+ pTau231	0.894	0.855	0.934	217
	Demographics + Aβ42/Aβ40 + GFAP + NfL + pTau181	0.895	0.859	0.932	219
TRIAD	pTau217	0.923	0.888	0.957	193
	pTau181	0.798	0.745	0.851	301
	GFAP	0.783	0.729	0.838	292
	Aβ42/Aβ40	0.736	0.676	0.797	324
	NfL	0.624	0.557	0.692	355
	Demographics + pTau217	0.929	0.896	0.961	193
	Demographics + Aβ42/Aβ40 + GFAP + pTau181	0.861	0.817	0.904	253
	Demographics + Aβ42/Aβ40 + GFAP + NfL + pTau181	0.862	0.819	0.906	254
	Demographics + Aβ42/Aβ40 + GFAP	0.840	0.793	0.888	265
	Demographics + Aβ42/Aβ40 + GFAP + NfL	0.841	0.794	0.888	266
	Demographics + Aβ42/Aβ40 + pTau181	0.841	0.794	0.888	268
SPIN	pTau217	0.957	0.924	0.990	69
	GFAP	0.904	0.854	0.953	103
	NfL	0.865	0.802	0.929	123
	pTau181	0.865	0.807	0.922	129
	pTau231	0.860	0.805	0.916	122
	Demographics + pTau217	0.969	0.931	1.000	59
	Demographics + GFAP + pTau231	0.940	0.887	0.994	82
	Demographics + pTau231	0.935	0.884	0.987	83
	Demographics + NfL + pTau231	0.940	0.887	0.993	83
	Demographics + GFAP	0.938	0.887	0.988	84
	Demographics + GFAP + NfL + pTau231	0.939	0.884	0.993	84

The table demonstrated the area under the curve (AUC) for plasma biomarkers and their combinations, in logistic regression models, and their associated 95% confidence intervals (CI) for predicting Aβ-positivity (defined with Aβ-PET in WRAP and TRIAD, and with CSF Aβ42/Aβ40 in SPIN). The Akaike information criterion (AIC) is also shown.

© 2024 Ashton NJ et al. *JAMA Neurology.*

eTable 5 – Receiver operating characteristics curves of plasma biomarker to determine tau positivity.

Cohort	Biomarker	AUC	CI Lower	CI Upper	AIC
WRAP	pTau217	0.927	0.845	1.000	71
	pTau181	0.855	0.767	0.944	118
	GFAP	0.837	0.755	0.918	116
	pTau231	0.800	0.707	0.894	117
	Aβ42/Aβ40	0.776	0.696	0.856	126
	NfL	0.719	0.611	0.826	133
	Demographics + Aβ42/Aβ40 + GFAP + pTau181+ pTau231	0.901	0.838	0.963	100
	Demographics + Aβ42/Aβ40 + GFAP + NfL + pTau181	0.903	0.842	0.964	101
	Demographics + Aβ42/Aβ40 + GFAP + NfL + pTau181+ pTau231	0.903	0.842	0.963	102
	Demographics + Aβ42/Aβ40 + pTau181+ pTau231	0.896	0.832	0.960	103
	Demographics + pTau217	0.941	0.880	1.000	71
	Demographics + Aβ42/Aβ40 + GFAP + pTau181	0.898	0.835	0.962	99
TRIAD	pTau217	0.946	0.920	0.973	156
	pTau181	0.855	0.804	0.906	210
	GFAP	0.851	0.796	0.905	200
	Aβ42/Aβ40	0.696	0.617	0.775	250
	NfL	0.696	0.621	0.771	262
	Demographics + pTau217	0.947	0.911	0.982	152
	Demographics + Aβ42/Aβ40 + GFAP + pTau181	0.920	0.885	0.955	162
	Demographics + Aβ42/Aβ40 + GFAP + NfL + pTau181	0.920	0.884	0.955	164
	Demographics + GFAP + pTau181	0.906	0.865	0.946	170
	Demographics + GFAP + NfL + pTau181	0.906	0.865	0.946	172
	Demographics + Aβ42/Aβ40 + GFAP	0.912	0.874	0.949	175
SPIN	pTau217	0.970	0.944	0.997	56
	GFAP	0.906	0.858	0.954	81
	pTau181	0.905	0.858	0.953	115
	pTau231	0.886	0.837	0.935	105
	NfL	0.834	0.764	0.904	126
	Demographics + pTau217	0.984	0.966	1.000	47
	Demographics + GFAP + NfL + pTau231	0.974	0.952	0.996	62
	Demographics + GFAP + pTau231	0.970	0.946	0.994	63
	Demographics + GFAP + NfL + pTau181+ pTau231	0.973	0.951	0.995	64
	Demographics + GFAP + pTau181+ pTau231	0.970	0.946	0.994	65
	Demographics + pTau231	0.961	0.929	0.992	68

The table demonstrated the area under the curve (AUC) for plasma biomarkers and their combinations, in logistic regression models, and their associated 95% confidence intervals (CI) for predicting tau-positivity (defined with tau-PET in WRAP and TRIAD, and with CSF pTau181 in SPIN). The Akaike information criterion (AIC) is also shown.

© 2024 Ashton NJ et al. *JAMA Neurology.*

eTable 6. Binary reference range for tau-positivity

	Binary reference-point for tau-positivity, Plasma pTau217 > 0.64 pg/mL		
	WRAP	TRIAD	SPIN
Number of participants	266	266	184
Tau positive, n (%)	19 (7.1)	55 (20.7)	103 (56.0)
Plasma pTau217 status positive, n (%)	49 (18.4)	82 (30.8)	99 (53.8)
Sensitivity, %	89.5	89.1	93.2
Specificity, %	87.0	84.4	96.3
PPA, %	34.7	59.8	97.0
NPA, %	99.1	96.7	91.8
OPA, %	87.0	85.3	94.6

The table shows key metrics for the evaluation of a binary ALZpath pTau217 reference-point for tau-positivity, derived in WRAP based in the Youden index, and directly cross-validated in TRIAD and SPIN. In WRAP and TRIAD, tau-positivity was determined with tau-PET, whereas in SPIN with CSF pTau181. PPA = positive percent agreement. NPA = negative percent agreement. OPA = overall percent agreement.

© 2024 Ashton NJ et al. *JAMA Neurology.*

eReferences

1. Therriault J, Benedet AL, Pascoal TA, et al. Association of Apolipoprotein E epsilon4 With Medial Temporal Tau Independent of Amyloid-beta. *JAMA Neurol*. Apr 1 2020;77(4):470-479. doi:10.1001/jamaneurol.2019.4421
2. Hansen TI, Brezova V, Eikenes L, Haberg A, Vangberg TR. How Does the Accuracy of Intracranial Volume Measurements Affect Normalized Brain Volumes? Sample Size Estimates Based on 966 Subjects from the HUNT MRI Cohort. *AJNR Am J Neuroradiol*. Aug 2015;36(8):1450-6. doi:10.3174/ajnr.A4299
3. Cselenyi Z, Jonhagen ME, Forsberg A, et al. Clinical validation of 18F-AZD4694, an amyloid-beta-specific PET radioligand. *J Nucl Med*. Mar 2012;53(3):415-24. doi:10.2967/jnumed.111.094029
4. Pascoal TA, Shin M, Kang MS, et al. In vivo quantification of neurofibrillary tangles with [(18)F]MK-6240. *Alzheimers Res Ther*. Jul 31 2018;10(1):74. doi:10.1186/s13195-018-0402-y
5. Pascoal TA, Therriault J, Benedet AL, et al. 18F-MK-6240 PET for early and late detection of neurofibrillary tangles. *Brain*. Sep 1 2020;143(9):2818-2830. doi:10.1093/brain/awaa180
6. Therriault J, Benedet AL, Pascoal TA, et al. Determining Amyloid-beta Positivity Using (18)F-AZD4694 PET Imaging. *J Nucl Med*. Feb 2021;62(2):247-252. doi:10.2967/jnumed.120.245209
7. Klunk WE, Koeppe RA, Price JC, et al. The Centiloid Project: standardizing quantitative amyloid plaque estimation by PET. *Alzheimers Dement*. Jan 2015;11(1):1-15 e1-4. doi:10.1016/j.jalz.2014.07.003
8. Therriault J, Pascoal TA, Benedet AL, et al. Frequency of Biologically Defined Alzheimer Disease in Relation to Age, Sex, APOE epsilon4, and Cognitive Impairment. *Neurology*. Feb 16 2021;96(7):e975-e985. doi:10.1212/WNL.0000000000011416
9. Ashton NJ, Benedet AL, Pascoal TA, et al. Cerebrospinal fluid p-tau231 as an early indicator of emerging pathology in Alzheimer's disease. *EBioMedicine*. Feb 11 2022;76:103836. doi:10.1016/j.ebiom.2022.103836
10. Ashton NJ, Pascoal TA, Karikari TK, et al. Plasma p-tau231: a new biomarker for incipient Alzheimer's disease pathology. *Acta Neuropathol*. May 2021;141(5):709-724. doi:10.1007/s00401-021-02275-6
11. Karikari TK, Pascoal TA, Ashton NJ, et al. Blood phosphorylated tau 181 as a biomarker for Alzheimer's disease: a diagnostic performance and prediction modelling study using data from four prospective cohorts. *Lancet Neurol*. May 2020;19(5):422-433. doi:10.1016/S1474-4422(20)30071-5
12. Johnson SC, Koscik RL, Jonaitis EM, et al. The Wisconsin Registry for Alzheimer's Prevention: A review of findings and current directions. *Alzheimers Dement (Amst)*. 2018;10:130-142. doi:10.1016/j.dadm.2017.11.007

© 2024 Ashton NJ et al. *JAMA Neurology.*

13. Johnson SC, Christian BT, Okonkwo OC, et al. Amyloid burden and neural function in people at risk for Alzheimer's Disease. *Neurobiol Aging*. Mar 2014;35(3):576-84. doi:10.1016/j.neurobiolaging.2013.09.028

14. Betthauser TJ, Koscik RL, Jonaitis EM, et al. Amyloid and tau imaging biomarkers explain cognitive decline from late middle-age. *Brain*. Jan 1 2020;143(1):320-335. doi:10.1093/brain/awz378

15. Betthauser TJ, Cody KA, Zammit MD, et al. In Vivo Characterization and Quantification of Neurofibrillary Tau PET Radioligand (18)F-MK-6240 in Humans from Alzheimer Disease Dementia to Young Controls. *J Nucl Med*. Jan 2019;60(1):93-99. doi:10.2967/jnumed.118.209650

16. Betthauser TJ, Bilgel M, Koscik RL, et al. Multi-method investigation of factors influencing amyloid onset and impairment in three cohorts. *Brain*. Nov 21 2022;145(11):4065-4079. doi:10.1093/brain/awac213

17. Jack CR, Jr., Wiste HJ, Weigand SD, et al. Defining imaging biomarker cut points for brain aging and Alzheimer's disease. *Alzheimers Dement*. Mar 2017;13(3):205-216. doi:10.1016/j.jalz.2016.08.005

18. Van Hulle C, Jonaitis EM, Betthauser TJ, et al. An examination of a novel multipanel of CSF biomarkers in the Alzheimer's disease clinical and pathological continuum. *Alzheimers Dement*. Mar 2021;17(3):431-445. doi:10.1002/alz.12204

19. Alcolea D, Clarimon J, Carmona-Iragui M, et al. The Sant Pau Initiative on Neurodegeneration (SPIN) cohort: A data set for biomarker discovery and validation in neurodegenerative disorders. *Alzheimers Dement (N Y)*. 2019;5:597-609. doi:10.1016/j.trci.2019.09.005

20. Iulita MF, Bejanin A, Vilaplana E, et al. Association of biological sex with clinical outcomes and biomarkers of Alzheimer's disease in adults with Down syndrome. *Brain Commun*. 2023;5(2):fcad074. doi:10.1093/braincomms/fcad074

21. Alcolea D, Pegueroles J, Munoz L, et al. Agreement of amyloid PET and CSF biomarkers for Alzheimer's disease on Lumipulse. *Ann Clin Transl Neurol*. Sep 2019;6(9):1815-1824. doi:10.1002/acn3.50873

© 2024 Ashton NJ et al. *JAMA Neurology.*
