# Supplementary material for: Diagnostic Accuracy of a Plasma Phosphorylated Tau 217 Immunoassay for Alzheimer Disease Pathology
Source: JAMA Neurol. 2024 Jan 22;81(3):255–63. doi: 10.1001/jamaneurol.2023.5319 (PMC10804282; doi:10.1001/jamaneurol.2023.5319)
Supplement: Supplement 2. — Data sharing statement [file jamaneurol-e235319-s002.pdf]

# Data Sharing Statement

Ashton. Diagnostic Accuracy of a Plasma Phosphorylated Tau 217 Immunoassay for Alzheimer Disease Pathology. *JAMA Neurol*. Published January 22, 2024.
doi:10.1001/jamaneurol.2023.5319

**Data**
**Data available:** No
